# Altered molecular and cellular mechanisms in *KIF5A*-associated neurodegenerative or neurodevelopmental disorders

**DOI:** 10.1038/s41419-024-07096-5

**Published:** 2024-09-27

**Authors:** Marta Cozzi, Stefania Magri, Barbara Tedesco, Guglielmo Patelli, Veronica Ferrari, Elena Casarotto, Marta Chierichetti, Paola Pramaggiore, Laura Cornaggia, Margherita Piccolella, Mariarita Galbiati, Paola Rusmini, Valeria Crippa, Jessica Mandrioli, Davide Pareyson, Chiara Pisciotta, Stefano D’Arrigo, Antonia Ratti, Lorenzo Nanetti, Caterina Mariotti, Elisa Sarto, Viviana Pensato, Cinzia Gellera, Daniela Di Bella, Riccardo M. Cristofani, Franco Taroni, Angelo Poletti

**Affiliations:** 1 Department of Pharmacological and Biomolecular Sciences “Rodolfo Paoletti” (DiSFeB), Università degli Studi di Milano, 20133 Milan, Italy; 2 Unit of Medical Genetics and Neurogenetics, Fondazione IRCCS Istituto Neurologico Carlo Besta, 20133 Milan, Italy; 3 Department of Biomedical, Metabolic and Neural Sciences, University of Modena and Reggio Emilia, Centre for Neuroscience and Neurotechnology (CfNN), 41125 Modena, Italy; 4 Department of Neurosciences, Azienda Ospedaliero-Universitaria di Modena, 41126 Modena, Italy; 5 Unit of Rare Neurological Diseases, Fondazione IRCCS Istituto Neurologico Carlo Besta, 20133 Milan, Italy; 6 Department of Pediatric Neurosciences, Fondazione IRCCS Istituto Neurologico Carlo Besta, 20133 Milan, Italy; 7 Department of Medical Biotechnology and Translational Medicine, Università degli Studi di Milano, 20054 Segrate, Italy; 8 Department of Neuroscience – Laboratory of Neuroscience, IRCCS Istituto Auxologico Italiano, 20095 Cusano Milanino, Italy

**Keywords:** Amyotrophic lateral sclerosis, Amyotrophic lateral sclerosis

## Abstract

Mutations targeting distinct domains of the neuron-specific kinesin KIF5A associate with different neurodegenerative/neurodevelopmental disorders, but the molecular bases of this clinical heterogeneity are unknown. We characterised five key mutants covering the whole spectrum of *KIF5A*-related phenotypes: spastic paraplegia (SPG, R17Q and R280C), Charcot-Marie-Tooth disease (CMT, R864*), amyotrophic lateral sclerosis (ALS, N999Vfs*40), and neonatal intractable myoclonus (NEIMY, C975Vfs*73) KIF5A mutants. CMT-R864*-KIF5A and ALS-N999Vfs*40-KIF5A showed impaired autoinhibition and peripheral localisation accompanied by altered mitochondrial distribution, suggesting transport competence disruption. ALS-N999Vfs*40-KIF5A formed SQSTM1/p62-positive inclusions sequestering WT-KIF5A, indicating a gain of toxic function. SPG-R17Q-KIF5A and ALS-N999Vfs*40-KIF5A evidenced a shorter half-life compared to WT-KIF5A, and proteasomal blockage determined their accumulation into detergent-insoluble inclusions. Interestingly, SPG-R280C-KIF5A and ALS-N999Vfs*40-KIF5A both competed for degradation with proteasomal substrates. Finally, NEIMY-C975Vfs*73-KIF5A displayed a similar, but more severe aberrant behaviour compared to ALS-N999Vfs*40-KIF5A; these two mutants share an abnormal tail but cause disorders on the opposite end of *KIF5A*-linked phenotypic spectrum. Thus, our observations support the pathogenicity of novel *KIF5A* mutants, highlight abnormalities of recurrent variants, and demonstrate that both unique and shared mechanisms underpin *KIF5A*-related diseases.

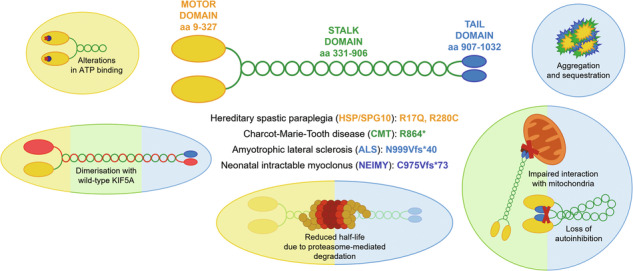

## Introduction

**Fig. 1:**
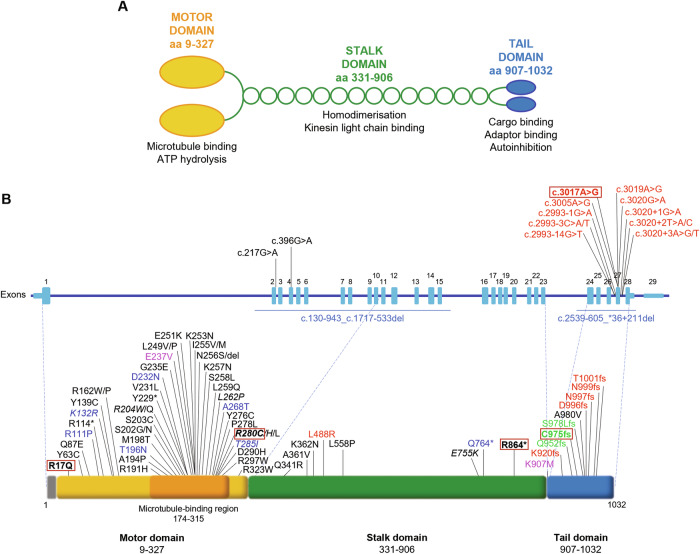
KIF5A structure and distribution of pathogenic variants. **A** Schematic representation of KIF5A structure, including KIF5A domains and their main functions. **B** Distribution of *KIF5A* pathogenic variants associated with HSP/SPG10 (black), CMT (blue), ALS (red), and NEIMY (green) phenotypes. The p.E237V and p.K907M variants (purple) are associated with West syndrome and severe global developmental delay, and Leber optic neuropathy, respectively. Variants associated with more than one disease are indicated in italics. The variants investigated in this study are indicated in bold and boxed. See Supplementary Table 1 for a detailed list of the variants and the associated phenotypes.

Kinesins are ATP-dependent molecular motors that transport cargoes towards microtubule plus-end participating in several cell functions, from intracellular trafficking to cell division [1]. The human kinesin family comprises more than 40 proteins divided into 15 subgroups. The three members of the KIF5 subfamily, KIF5A, KIF5B, and KIF5C, were the first ones to be identified. While KIF5B is ubiquitously expressed, KIF5A and KIF5C are neuron-specific [2]. KIF5s share a common structure comprising an N-terminal motor head, a central coiled-coil stalk, and a C-terminal globular tail (Fig. 1A). The head domain binds microtubules and fuels transport through ATP hydrolysis. The stalk domain mediates KIF5 homodimerisation, conformational changes, and interaction with kinesin light chains. The tail domain is involved in cargo/adaptor binding, microtubule sliding/bundling, and autoinhibition [1]. Autoinhibition prevents unnecessary kinesin movements in the absence of cargo and is achieved through direct interaction between the KIF5 motor domain and an isoleucine-alanine-lysine (IAK) motif in the tail. This state is relieved upon interaction with cargoes or adaptors, that promotes KIF5 unfolding and stepping along microtubules [3]. KIF5s mediate the anterograde axonal transport of several cargoes, including proteins, RNA granules, and organelles [4–8].

In the past 20 years, numerous disease-causing variants have been identified in the *KIF5A* gene (Fig. 1B and Supplementary Table 1). Heterozygous missense variants in the motor and stalk domains are associated with spastic paraplegia type 10 (SPG10, Mendelian Inheritance in Man (MIM) #604187), a form of hereditary spastic paraplegia (HSP) [9], and axonal Charcot-Marie-Tooth disease (CMT) [10]. Frameshift mutations targeting the KIF5A tail are instead linked to ALS (MIM#617921) [11, 12] and to a complex infantile neurodevelopmental disorder with leukoencephalopathy named neonatal intractable myoclonus (NEIMY, MIM#617235) [13, 14]. To date, the molecular mechanisms whereby *KIF5A* mutations lead to neurodegeneration have been only partially characterised. Generally, variants in the motor domain impair microtubule binding and/or ATP hydrolysis, resulting in reduced KIF5A motility and/or anterograde transport [15]. On the other hand, mutations leading to tail elongation associated or not with exon 27 skipping (ΔExon27) abolish KIF5A autoinhibition and cause protein aggregation and limited cytoplasmic recycling, accompanied by WT KIF5A sequestration into inclusions [16–18]. Despite these findings, the exact pathogenic mechanisms whereby mutations in different KIF5A domains give rise to distinct phenotypes are yet to be resolved.

Here, we studied in our series of patients and selected for functional characterisation four variants associated with different *KIF5A* phenotypes—SPG10, CMT, and ALS—to gain insight into the unique and shared molecular mechanisms driving *KIF5A*-related pathologies. Based on the observations made on the ALS-associated ΔExon27 p.N999Vfs*40 KIF5A mutant [11, 12], we then compared its behaviour with that of p.C975Vfs*73 KIF5A [14], a NEIMY-linked variant that shares its elongated tail.

## Results

### Patients and *KIF5A* variants

Between 2008 and 2022, the *KIF5A* gene was analysed in ~2150 index cases referred for genetic testing to the Istituto Neurologico Carlo Besta. Clinical diagnosis was HSP in ~600 of them, CMT in ~700, ALS in ~400, and developmental epileptic encephalopathy (DEE) in ~450. Heterozygous pathogenic or likely pathogenic variants [19] were identified in 29 cases (19 HSP, 9 CMT, 1 ALS). In addition, novel missense variants of uncertain significance (VUS) located in the motor domain were found in four probands with a spastic paraplegia phenotype. No variants were found in the DEE group, where NEIMY patients are expected to be included.

**Fig. 2:**
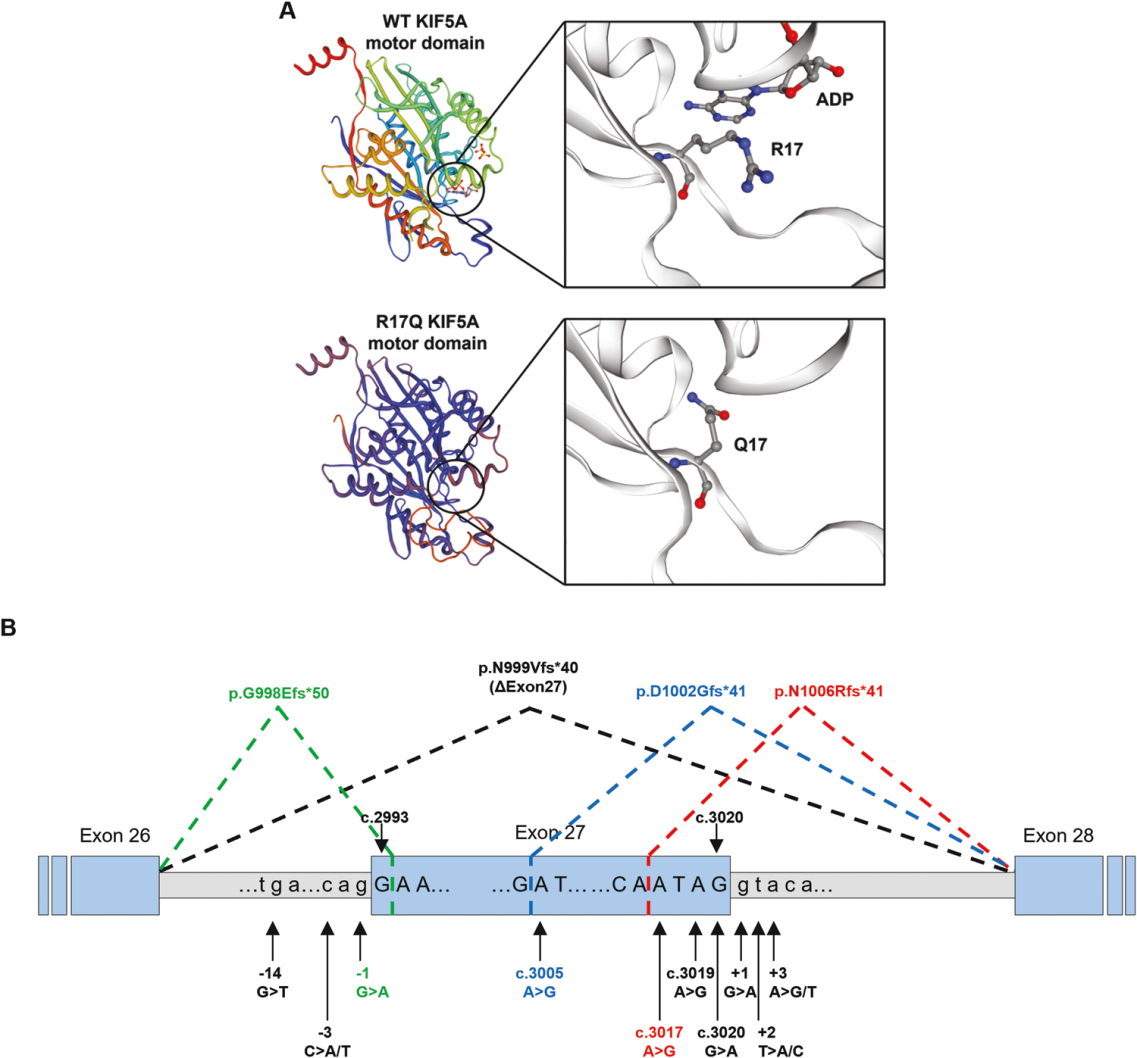
Predicted effects of the novel variants p.R17Q and c.3017 A > G. **A** p.R17Q KIF5A motor domain structure modelled by SWISS-MODEL (https://swissmodel.expasy.org) using wild-type (WT) KIF5A head as template (SMTL ID: 1mkj.1). Modelling predicts the loss of the ATP/ADP binding site in KIF5A motor domain as a consequence of the R17Q substitution. **B** Splicing prediction of variants identified in exon 27 and intron flanking regions. Diagram of the effect (either predicted or experimentally proven) of different splicing variants. The dotted lines indicate the probable aberrant effect on splicing based on the algorithms used. Variants that cause skipping of exon 27 (p.N999Vfs*40, ΔExon27) are indicated in black, the c.2993-1 G > A predicted to result in p.G998Efs*50 is in green, and the c.3005 A > G predicted to cause p.D1002Gfs*41 is in blue. The novel variant c.3017 A > G reported in this study is indicated in red.

For this study, we decided to compare the functional effects of the recurrent p.R280C variant, which was the most frequent in our series (7/29 index cases, Supplementary Table 2), the nonsense mutation p.R864*, previously reported in an HSP patient [20] and found by us in a CMT2 patient, the NEIMY-associated variant p.C975Vfs*73, the ALS-associated p.N999Vfs*40, and the c.50 G > A/p.R17Q [21], one of the 4 novel VUS identified in our series. This mutation lies in the motor domain and may represent the most N-terminal pathogenic variant in KIF5A. Although it is classified as VUS according to the American College of Medical Genetics and Genomics (ACMG) criteria [19], its potential pathological relevance is supported by the following points: (a) it is absent in population databases including gnomAD, TOPMed, 1000 Genomes (PM2); (b) it co-segregates with the disease in an autosomal dominant pattern (PP1; Supplementary Table 2); (c) the *KIF5A* gene is intolerant to missense variants (gnomAD missense constraint z-score = 3.6; PP2); (d) in silico aggregated scores predict a deleterious effect (REVEL [22]: Supporting pathogenic (0.74), CADD [23]: 29.4) and the variant affects a conserved residue in a conserved region of the protein (PP3; Supplementary Fig. 1). Interestingly, structural modelling shows that the arginine-17 residue lies in the ATP-binding pocket of the KIF5A motor domain and predicts that the p.R17Q substitution would hamper ATP/ADP binding (Fig. 2A), thus altering the ability of mutant KIF5A to fuel transport.

In the ALS series, only one patient carried a *KIF5A* variant, a novel c.3017 A > G/p.N1006S substitution classified as likely pathogenic according to the ACMG criteria [19]: it is absent in population databases (gnomAD, TOPMed, 1000 Genomes (PM2)), and, most importantly, it is predicted by different tools to cause aberrant splicing (PP3). This variant lies in exon 27, that is strongly associated with ALS phenotypes [12] (Supplementary Table 1) and is predicted to attenuate/abolish the constitutive donor site of intron 27 and create a new GT donor site within exon 27 at position c.3017_3018 (Fig. 2B and Table 1). This would generate two possible transcripts, the recurrent form ΔExon27 (r.2993_3020del, p.N999Vfs*40) [11, 12, 16, 18], and/or a novel frameshift form retaining part of exon 27 (r.(3017_3020del), p.(N1006Rfs*41)). The effects on the KIF5A protein are expected to be similar, as both transcripts would encode a common C-terminal tail (Supplementary Fig. 2). Unfortunately, the splicing effects could not be assessed at the mRNA level due to the lack of biological samples from this patient.

**Table 1:** In silico splicing prediction of variants identified in exon 27 and intron flanking regions.

	Splicing prediction				
Variant (predicted protein effect)	Alternative Splice Site Predictor	NNSplice	SpliceAI	dbscSNV Ada	dbscSNV RF
c.2993-14 G > T (p.N999Vfs*40)	cAS 6.535 → 8.358	cAS 0.49 → 0.70	Splice-Altering (low) (0.22)	n.a.	n.a.
c.2993-3 C > A (p.N999Vfs*40)	cAS 6.535 → 2.885	cAS 0.49→abolished	Splice-Altering (low) (0.26)	Deleterious (1)	Deleterious (0.98)
c.2993-3 C > T (p.N999Vfs*40)	cAS 6.535 → 5.216	cAS 0.49→abolished	Splice-Altering (low) (0.43)	Deleterious (0.95)	Benign (0.32)
c.2993-1 G > A (p.G998Efs*50)	cAS 6.535→abolished; new AS c.2994 (2.114)	cAS 0.49→abolished	Splice-Altering (0.9)	Deleterious (1)	Deleterious (0.94)
c.3005 A > G (p.D1002Gfs*41)	cDS 8.658→new DS c.3004 (10.814)	cDS 0.79→abolished new DS c.3005 (0.81)	Splice-Altering (0.61)	n.a.	n.a.
*c.3017* *A* > G (p.N1006Rfs*41)	*cDS 8.658* → *7.932; new DS c.3016 (8.440)*	*cDS 0.79→abolished; new DS c.3017 (0.65)*	*Splice-Altering (0.90)*	*n.a*.	*n.a*.
c.3019 A > G (p.N999Vfs*40)	cDS 8.658 → 5.234	cDS 0.79 → 0.16	Splice-Altering (low) (0.49)	Deleterious (1)	Deleterious (1)
c.3020 G > A (p.N999Vfs*40)	cDS 8.658 → 5.119	cDS 0.79→abolished	Splice-Altering (0.66)	Deleterious (1)	n.a.
c.3020+1 G > A (p.N999Vfs*40)	cDS 8.658→abolished	cDS 0.79→abolished	Splice-Altering (0.69)	Deleterious (1)	Deleterious (0.95)
c.3020+2 T > A (p.N999Vfs*40)	cDS 8.658→abolished	cDS 0.79→abolished	Splice-Altering (0.69)	Deleterious (1)	Deleterious (0.95)
c.3020+2 T > C (p.N999Vfs*40)	cDS 8.658→abolished	cDS 0.79→abolished	Splice-Altering (0.69)	Deleterious (1)	Deleterious (0.82)
c.3020+3 A > T (p.N999Vfs*40)	cDS 8.658 → 4.436	cDS 0.79→abolished	Splice-Altering (0.6)	Deleterious (1)	Deleterious (0.88)
c.3020+3 A > G (p.N999Vfs*40)	cDS 8.658 → 6.15	cDS 0.79 → 0.14	Splice-Altering (0.68)	Deleterious (1)	Deleterious (0.97)

The novel variant c.3017 A > G reported in this study is indicated in italics

*AS* splicing acceptor site, *cAS* constitutive splicing acceptor site, *cDS* constitutive splicing donor site, *DS* splicing donor site, n.a. not available.

References for the reported variants can be found in Supplementary Table 1.

### Clinical phenotypes of selected *KIF5A* mutation carriers

The detailed clinical and instrumental findings on the patients carrying the selected *KIF5A* variants are described in Supplementary Table 2. There were seven women and six men. Autosomal dominant family history was reported in 3/10 families (six patients), two patients were adopted, and the remaining five subjects had a sporadic presentation. Age at onset was >34 years in the family with the p.R17Q variant and <30 years in patients with the p.R280C variant (range 2–29 years). Spastic paraplegia was the predominant phenotype in most patients with a variant in the motor domain (9/11, Patients 1–6, 8–10). Seven of them also exhibited axonal polyneuropathy (Patients 1, 2, 4, 5, 7, 10, 11). Two patients with the p.R280C variant presented with a CMT5 phenotype, an autosomal dominant form of axonal CMT with pyramidal involvement (Patients 7 and 11). The patient with the p.R864* variant (Patient 12) presented three episodes of Parsonage-Turner syndrome in upper limbs at age 14, 16, and 20 and axonal sensory neuropathy in lower limbs at age 20. The patient carrying the p.(N1006R) variant (Patient 13) was diagnosed with ALS at the age of 60 after a 12-month history of right arm muscle weakness and atrophy, and widespread cramps. Family history was negative. Neurological examination showed upper limb weakness with hypotrophy and widespread fasciculations, marked lower limb hyperreflexia, and right Babinski sign. Respiratory function was normal (forced vital capacity 87%), and the revised ALS functional rating scale [24] score was 37/48. Disease progression was fast and characterised by rapidly worsening four limbs weakness, widespread upper and lower motor neuron signs, and bulbar and respiratory involvement, which occurred 5 months after diagnosis. The patient accepted non-invasive ventilation but refused other supports and died from respiratory insufficiency 3 months later (20 months after symptoms onset).

### KIF5A mutants display altered localisation, levels, and solubility

**Fig. 3:**
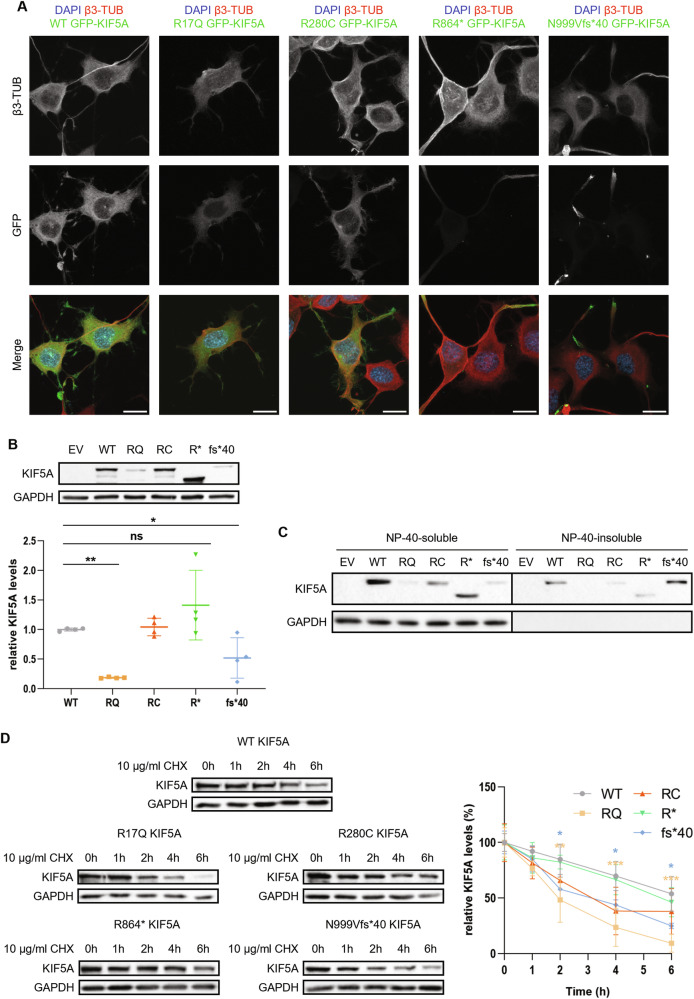
Mutant KIF5A localisation, levels, and solubility. **A** Confocal microscopy analysis (63× magnification) of NSC-34 cells transiently transfected with WT or mutant pGFP-KIF5A constructs. Endogenous β3-tubulin was stained in red. Nuclei were stained with DAPI. Scale bar 20 µm. **B** Western blot analysis of KIF5A protein levels in SH-SY5Y cells transiently transfected with WT or mutant pKIF5A constructs (*N* = 4). An empty vector (EV) was used as a transfection mock. GAPDH protein levels were used for normalisation. The graph represents mean optical densities relative to samples overexpressing WT KIF5A ± SD. One-way ANOVA with Fisher’s LSD post-test was performed. ns not significant; *P < 0.05; **P < 0.01. **C** Western blot analysis of KIF5A fractionation between the NP-40–soluble and the NP-40–insoluble protein fractions deriving from the same whole cell lysate of SH-SY5Y cells transiently transfected with WT or mutant pKIF5A constructs. **D** Western blot analysis of KIF5A protein levels in SH-SY5Y cells transiently transfected with WT or mutant pKIF5A constructs and treated with 10 µg/ml CHX for 1-2-4-6 h (*N* = 3). GAPDH protein levels were used for normalisation. The graph represents mean optical densities expressed as percentages of either WT or mutant KIF5A baseline (i.e., 0 h) levels ± SD. Two-way ANOVA with Sidak’s post-test was performed comparing WT and mutant KIF5A protein levels at each time point. *P < 0.05; **P < 0.01; ***P < 0.001.

We initially characterised the intracellular distribution of the selected variants (hereafter: R17Q, R280C, R864*, N999Vfs*40, or C975Vfs*73 KIF5A) in NSC-34 cells. As expected, wild-type (WT) KIF5A appeared diffused across the cytoplasm, including neurites. The SPG10-related R17Q and R280C KIF5A mutants showed a similar distribution (Fig. 3A), even if the latter also accumulated into perinuclear puncta in a few cells (Supplementary Fig. 3A). Conversely, both the CMT-linked R864* and the ALS-associated N999Vfs*40 KIF5A mutants preferentially localised within neurites, but with a distinct distribution pattern: the R864* mutant appeared diffused in neurites, while N999Vfs*40 KIF5A formed inclusions at neurite tips (Fig. 3A and quantification in Supplementary Fig. 3B). The intracellular distribution found for the R864* and N999Vfs*40 KIF5A mutants is consistent with the impairment of kinesin autoinhibitory function, respectively depending on the loss and the modification of the KIF5A tail [3]. For the ALS-linked N999Vfs*40 KIF5A mutant, this abnormal protein distribution has already been associated with accumulation at microtubule plus-ends [16–18].

In parallel, we compared WT and mutant KIF5A protein levels upon SH-SY5Y cell transfection. While WT and R280C KIF5A levels were similar, significantly lower protein levels were detected for the R17Q and N999Vfs*40 mutants and a tendency to display higher protein levels was found for the R864* mutant with respect to WT KIF5A (Fig. 3B). We also tested KIF5A solubility by fractionating proteins in the mild detergent NP-40. As shown in Fig. 3C, N999Vfs*40 KIF5A was enriched in the insoluble protein fraction, differently from the other KIF5A mutants under analysis and the WT protein, that all resulted largely soluble in NP-40. This is in line with the aggregation-prone behaviour observed in immunofluorescence and suggests a potential gain of toxic function for N999Vfs*40 KIF5A. Since the RT-qPCR analysis showed comparable *KIF5A* mRNA levels between cells overexpressing WT or mutant KIF5A (Supplementary Fig. 3C), we hypothesised that the observed changes in KIF5A protein levels may depend on an altered turnover of the R17Q and N999Vfs*40 mutants compared to that of WT KIF5A. This was confirmed by cycloheximide (CHX) chase assay: overexpressed WT KIF5A displayed a ~6 h half-life in CHX-treated cells, while the protein levels of the R17Q and N999Vfs*40 KIF5A mutants were respectively reduced to ~10 and ~25% in the same time interval. On the contrary, WT, R280C, and R864* KIF5A showed comparable degradation profiles (Fig. 3D). Therefore, we observed a reduction in protein levels caused by protein instability and potentially restricting the pool of KIF5A motors available for anterograde transport for R17Q and N999Vfs*40 KIF5A, but not for the other mutants included in our study.

### Mutant KIF5A interaction with WT KIF5A and mitochondria

**Fig. 4:**
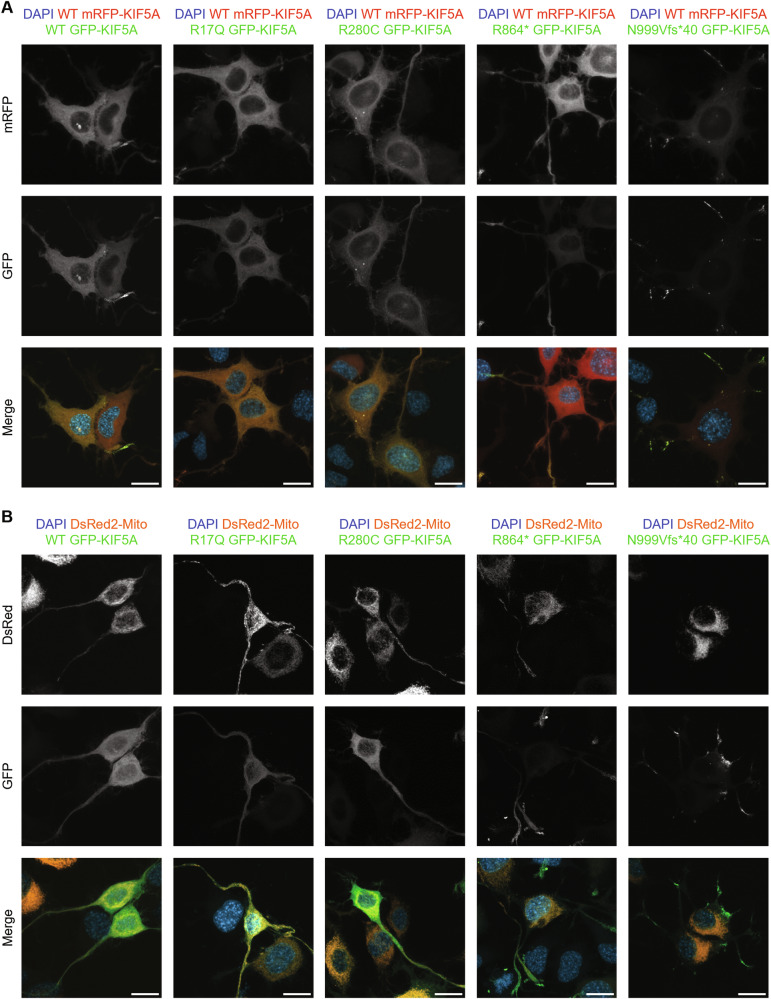
Reciprocal distribution between mutant KIF5A and the WT protein or KIF5A cargoes. **A** Confocal microscopy analysis (63× magnification) of NSC-34 cells transiently co-transfected with equal amounts of WT or mutant pGFP-KIF5A and WT pmRFP-KIF5A constructs. Nuclei were stained with DAPI. Scale bar 20 µm. **B** Confocal microscopy analysis (63× magnification) of NSC-34 transiently co-transfected with WT or mutant pGFP-KIF5A constructs and pDsRed2-Mito. Nuclei were stained with DAPI. Scale bar 20 µm.

In neurons, KIF5A works as a homodimer [25]. To assess whether the investigated KIF5A mutants were capable of dimerising with the WT protein, we co-transfected NSC-34 cells with equal amounts of WT pmRFP-KIF5A and pGFP-KIF5A constructs to test for WT and mutant KIF5A reciprocal distribution. As shown in Fig. 4A, the WT protein co-distributed with all KIF5A mutants. Notably, both R864* and N999Vfs*40 KIF5A partially sequestered the WT protein to the cell periphery (quantification in Supplementary Fig. 4A), a finding in line with previous reports on ΔExon27 KIF5A variants [16–18]. The interaction between WT and mutant KIF5A was also confirmed by co-immunoprecipitation in SH-SY5Y cells (Supplementary Fig. 4B). Therefore, these results suggest a possible dominant-negative effect of R864* and N999Vfs*40 KIF5A on the WT protein.

KIF5A transports mitochondria along axonal and dendritic processes [6, 7] and proper mitochondrial distribution and metabolism are essential to ensure neuronal homoeostasis. Based on these premises, we evaluated the reciprocal distribution of mitochondria and mutant KIF5A by co-transfecting NSC-34 cells with the mitochondrial reporter pDsRed2-Mito and pGFP-KIF5A constructs. Upon overexpression of R864* or N999Vfs*40 KIF5A, just a small fraction of mitochondria co-distributed with mutant KIF5A within neurites and very few organelles were found close to N999Vfs*40 KIF5A aggregates; instead, R17Q and R280C KIF5A showed a reciprocal distribution with their cargo similar to that of the WT protein (Fig. 4B). This observation hints that the R864* and N999Vfs*40 KIF5A mutants might display a defective interaction with the mitochondrion, with potentially pathogenic effects. Indeed, mitochondrial dysfunction has been associated with several neurodegenerative diseases, including *KIF5A*-related conditions [26]. Notably, a similar alteration in mitochondrial distribution was reported in an ALS-KIF5A *Drosophila* model [27].

### KIF5A mutants do not alter the basal autophagy flux

**Fig. 5:**
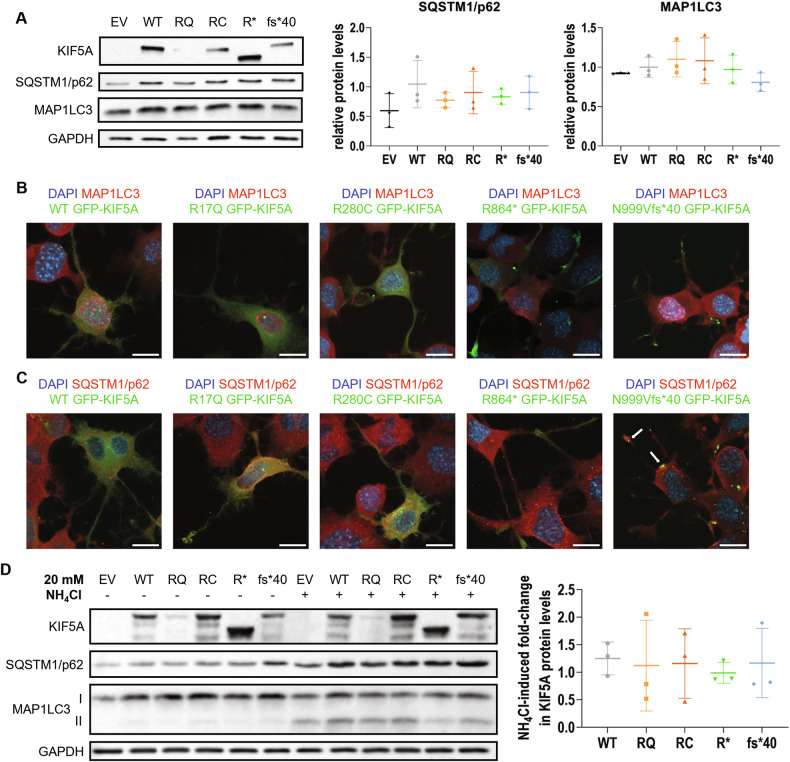
Interplay between mutant KIF5A and autophagy. **A** Western blot analysis of basal SQSTM1/p62 and MAP1LC3 protein levels in SH-SY5Y cells transiently transfected with WT or mutant pKIF5A constructs (*N* = 3). An empty vector (EV) was used as a transfection mock. GAPDH protein levels were used for normalisation. Graphs represent mean SQSTM1/p62 (left graph) and MAP1LC3 (right graph) optical densities relative to samples overexpressing WT KIF5A ± SD. One-way ANOVA with Fisher’s LSD post-test was performed. **B** Confocal microscopy analysis (63× magnification) of NSC-34 cells transiently transfected with WT or mutant pGFP-KIF5A constructs. Endogenous MAP1LC3 was stained in red. Nuclei were stained with DAPI. Scale bar 20 µm. **C** Confocal microscopy analysis (63× magnification) of NSC-34 cells transiently transfected with WT or mutant pGFP-KIF5A constructs. Endogenous SQSTM1/p62 was stained in red. Nuclei were stained with DAPI. Arrows highlight co-localisation between N999Vfs*40 KIF5A and SQSTM1/p62. Scale bar 20 µm. **D** Western blot analysis of KIF5A protein levels in SH-SY5Y cells transiently transfected with WT or mutant pKIF5A constructs and treated with 20 mM NH_4_Cl for 16 h (*N* = 3). The graph represents the mean fold-change of GAPDH-normalised WT or mutant KIF5A protein levels induced by the treatment ± SD. One-way ANOVA with Fisher’s LSD post-test was performed.

KIF5s participate in lysosomal transport and autophagy [8, 28]. The latter is one of the main protein degradation pathways, and its dysfunction is often involved in the pathogenesis of neurodegenerative diseases, including HSP and ALS [29, 30]. Since we observed an enhanced degradation rate and/or an aggregation-prone behaviour for some of the KIF5A mutants under investigation (Fig. 3), we analysed the possible interplay between mutant KIF5A and autophagy. We initially evaluated the basal protein levels of two autophagy markers, SQSTM1/p62 and MAP1LC3 [31], in KIF5A-overexpressing cells. Both SQSTM1/p62 and MAP1LC3 protein levels remained unchanged in all tested conditions in western blot (Fig. 5A) and immunofluorescence analysis showed no alterations in endogenous MAP1LC3 localisation (Fig. 5B). SQSTM1/p62 distribution, too, was unaltered, except for N999Vfs*40 KIF5A-overexpressing cells, in which mutant KIF5A inclusions resulted positive for this autophagy receptor (Fig. 5C). The direct interaction between N999Vfs*40 KIF5A and SQSTM1/p62 was confirmed by co-immunoprecipitation (Supplementary Fig. 4B). This aberrant interaction is consistent with previous reports [16, 18] and may indicate that the aggregation-prone, short-lived N999Vfs*40 KIF5A mutant is targeted to autophagy-mediated degradation by SQSTM1/p62. However, by treating KIF5A-overexpressing cells with the autophagosome-lysosome fusion inhibitor NH_4_Cl to assess the impact of autophagy blockage on KIF5A turnover, we did not observe any significant alteration in KIF5A protein levels and distribution, despite the expected increase in SQSTM1/p62 and MAP1LC3-II protein levels that confirms autophagy inhibition (Fig. 5D and Supplementary Fig. 5A).

Altogether, these results indicate that mutant KIF5A overexpression does not alter basal autophagy, that in turn does not represent the preferential degradation route for WT and mutant KIF5A, despite the abnormal interaction observed between SQSTM1/p62 and the ALS-linked N999Vfs*40 KIF5A mutant.

### WT and mutant KIF5A degradation is mainly mediated by the ubiquitin-proteasome system

**Fig. 6:**
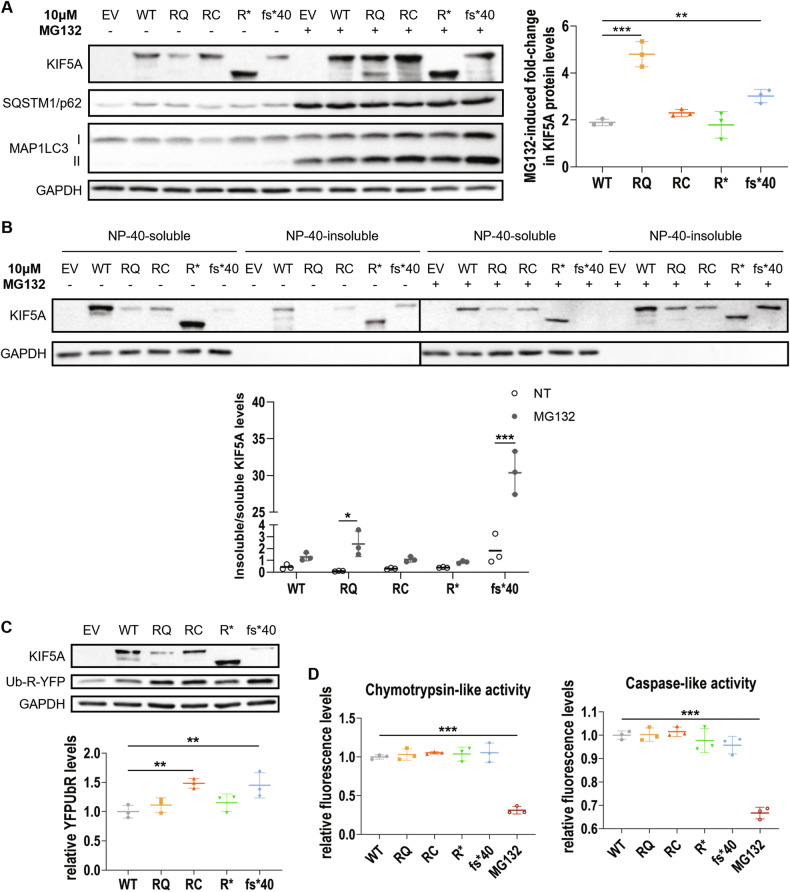
Interplay between mutant KIF5A and the UPS. **A** Western blot analysis of KIF5A protein levels in SH-SY5Y cells transiently transfected with WT or mutant pKIF5A constructs and treated with 10 µM MG132 for 16 h (*N* = 3). An empty vector (EV) was used as a transfection mock. The graph represents the mean fold-change of GAPDH-normalised WT or mutant KIF5A protein levels induced by the treatment ± SD. One-way ANOVA with Fisher’s LSD post-test was performed. *P < 0.05; ***P < 0.001. **B** Western blot analysis of KIF5A fractionation between the NP-40–soluble and the NP-40–insoluble protein fractions deriving from the same whole cell lysate of SH-SY5Y transiently transfected with WT or mutant pKIF5A constructs and treated with 10 µM MG132 for 16 h (*N* = 3). The graph represents mean ratio between KIF5A insoluble and soluble protein levels in each experimental condition ± SD. Two-way ANOVA with Fisher’s LSD post-test was performed comparing insoluble/soluble KIF5A ratio between untreated (NT) and treated (MG132) samples for each KIF5A condition. *P < 0.05; ***P < 0.001. **C** Western blot analysis of Ub-R-YFP protein levels in SH-SY5Y transiently co-transfected with WT or mutant pKIF5A constructs and Ub-R-YFP (*N* = 3). GAPDH protein levels were used for normalisation. The graph represents mean Ub-R-YFP optical densities relative to samples overexpressing WT KIF5A ± SD. One-way ANOVA with Fisher’s LSD post-test was performed. **P < 0.01. **D** Proteasome activity analysis in SH-SY5Y cells overexpressing WT or mutant pKIF5A constructs (*N* = 3). Specific AMC-conjugated peptides (N-Suc-LLVY-AMC and Z-LLE-AMC) were used to evaluate chymotrypsin-like (top graph) and caspase-like (bottom graph) proteasome activities. Samples treated with 1 µM MG132 for 16 h were used as control. Graphs represent mean fluorescence levels relative to samples overexpressing WT KIF5A ± SD. One-way ANOVA with Fisher’s LSD post-test was performed. *P < 0.05; ***P < 0.001.

We then tested whether the enhanced protein turnover observed for R17Q and N999Vfs*40 KIF5A (Fig. 3D) could depend on the activity of the other main protein degradation route in cells, the ubiquitin-proteasome system (UPS). Notably, SQSTM1/p62 is a ubiquitin-binding protein acting both as an autophagy receptor [31] and in the delivery of ubiquitinated substrates to the UPS by direct interaction with the 26S proteasome [32]. SQSTM1/p62 co-localisation with the aggregation-prone N999Vfs*40 KIF5A mutant could, therefore, be indicative of its targeting to the UPS instead of autophagy. To test this hypothesis, we treated KIF5A-overexpressing SH-SY5Y cells with the 26S proteasome inhibitor MG132 and observed an increase in protein levels for both WT and mutant KIF5A upon treatment. Interestingly, MG132-induced accumulation (i.e. fold-change of GAPDH-normalised KIF5A protein levels induced by the treatment) resulted statistically higher for the short-lived R17Q and N999Vfs*40 KIF5A mutants than for the WT protein (Fig. 6A). Moreover, proteasomal blockage induced the preferential partitioning of both R17Q and N999Vfs*40 KIF5A in the NP-40–insoluble protein fraction. Such transitioning was particularly relevant for the R17Q mutant, which appeared completely NP-40–soluble in basal conditions, and for the N999Vfs*40 mutant (Fig. 6B). Immunofluorescence analysis of NSC-34 cells treated with MG132 showed distribution into abundant perinuclear puncta for both R17Q and N999Vfs*40 KIF5A upon UPS blockage, differently from the other KIF5A mutants under analysis and from the WT protein, that remained largely diffused (Supplementary Fig. 5B). These results strongly suggest that R17Q and N999Vfs*40 KIF5A form potentially harmful inclusions in neurons when proteostasis is impaired.

To further investigate the interplay between KIF5A and the UPS, we co-expressed the proteasome activity reporter Ub-R-YFP [33] and pKIF5A constructs in SH-SY5Y cells and observed that Ub-R-YFP significantly accumulated upon concomitant overexpression of either R280C or N999Vfs*40 KIF5A compared to cells overexpressing the WT protein (Fig. 6C), suggesting that both KIF5A mutants might have a direct impact on the 26S proteasome activity. Ub-R-YFP accumulation in cells may occur either through proteasome inhibition or through competition between substrates destined for proteasomal degradation. To test which was the case upon mutant KIF5A overexpression, we assayed the enzymatic activity of the proteasome in KIF5A-overexpressing cells. In detail, we measured the conversion rate of the 7-amino-4-methyl coumarin (AMC)-conjugated substrates *N*-Suc-LLVY-AMC and Z-LLE-AMC, respectively targeted by the chymotrypsin-like and the caspase-like activities of the 26S proteasome. We found that the UPS was not inhibited by WT or mutant KIF5A overexpression (Fig. 6D). Thus, R280C and N999Vfs*40 KIF5A might compete with Ub-R-YFP for proteasome-mediated degradation, resulting in its accumulation in cells.

Taken together, our observations indicate that WT and mutant KIF5A turnover mainly depends on the UPS.

### NEIMY-KIF5A displays similar, but more severe defects compared to ALS-KIF5A

Frameshift mutations in the KIF5A tail domain have been associated with both ALS and NEIMY. Although these diseases are very different from one another, the underpinning *KIF5A* mutations are expected to produce partially overlapping effects on the corresponding protein [13, 14]. Based on the results obtained in N999Vfs*40 KIF5A characterisation, we compared its behaviour with that of the NEIMY-linked C975Vfs*73 KIF5A variant [14]. The latter presents an elongated tail in comparison to WT KIF5A and shares the last 40 amino acids with the N999Vfs*40 mutant, despite not being affected by exon 27 skipping (Supplementary Fig. 6). Interestingly, CamSol analysis [34] confirmed the low intrinsic solubility of the 40-residue tail common to N999Vfs*40 and C975Vfs*73 KIF5A, but also revealed the presence of a poorly soluble sequence in the C975Vfs*73 mutant that is missing in both WT and N999Vfs*40 KIF5A. Remarkably, such sequence corresponds to the amino acids encoded by *KIF5A* exon 27 (Supplementary Fig. 7). This would indicate an even lower solubility of C975Vfs*73 KIF5A with respect to N999Vfs*40 KIF5A. Notably, two other NEIMY-linked *KIF5A* mutations reported in the literature, c.2854delC/p.Q952Rfs*96 and c.2934delG/p.S978Vfs*70 KIF5A [13], share the frame and the low-solubility sequences in their aberrant tail with C975Vfs*73 KIF5A (Supplementary Figs. 6, 7), suggesting potential similarities in the behaviour of these variants.

**Fig. 7:**
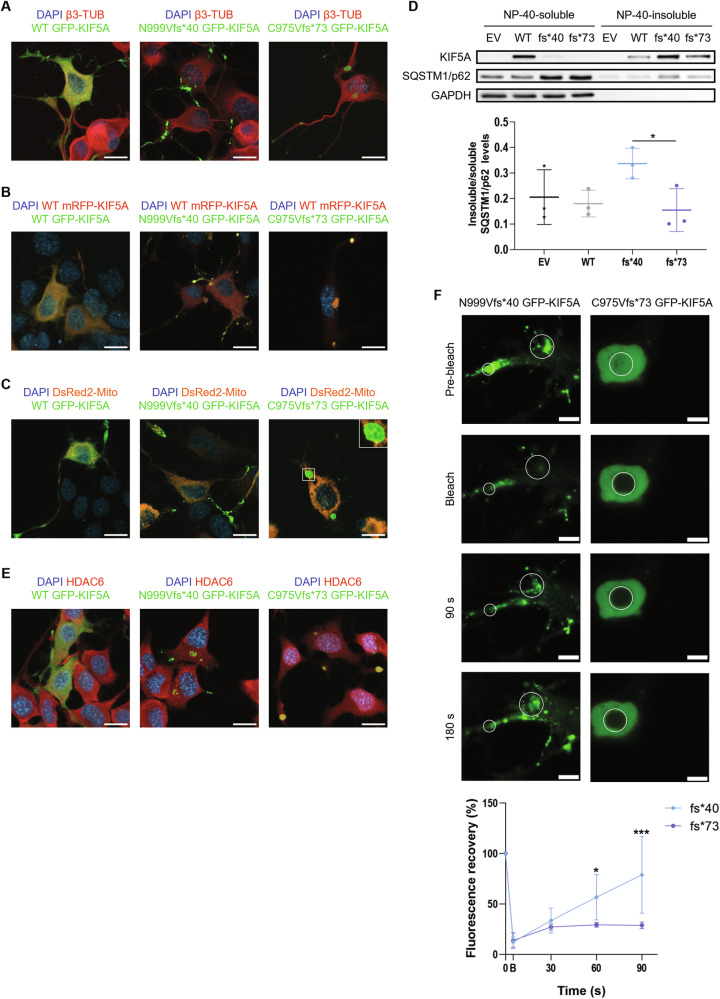
ALS- and NEIMY-KIF5A. **A** Confocal microscopy analysis (63× magnification) of NSC-34 cells transiently transfected with WT or frameshift pGFP-KIF5A constructs. Endogenous β3-tubulin was stained in red. Nuclei were stained with DAPI. Scale bar 20 µm. **B** Confocal microscopy analysis (63× magnification) of NSC-34 cells transiently co-transfected with equal amounts of WT or frameshift pGFP-KIF5A and WT pmRFP-KIF5A constructs. Nuclei were stained with DAPI. Scale bar 20 µm. **C** Confocal microscopy analysis (63× magnification) of NSC-34 cells transiently co-transfected with WT or frameshift pGFP-KIF5A constructs and pDsRed2-Mito. Nuclei were stained with DAPI. Scale bar 20 µm. **D** Western blot analysis of KIF5A and SQSTM1/p62 fractionation between the NP-40–soluble and the NP-40–insoluble protein fraction in SH-SY5Y cells transiently transfected with WT or frameshift pKIF5A constructs (*N* = 3). An empty vector (EV) was used as a transfection mock. The graph represents mean ratio between SQSTM1/p62 insoluble and soluble protein levels in each experimental condition ± SD. Student’s *t*-test was performed comparing SQSTM1/p62 insoluble/soluble ratio in samples overexpressing N999Vfs*40 or C975Vfs*73 KIF5A. *P < 0.05. **E** Confocal microscopy analysis (63× magnification) of NSC-34 cells transiently transfected with WT or frameshift pGFP-KIF5A constructs. Endogenous HDAC6 was stained in red. Nuclei were stained with DAPI. Scale bar 20 µm. **F** Fluorescence recovery after photobleaching analysis of frameshift KIF5A aggregates in NSC-34 cells 48 h after transient transfection with pGFP-KIF5A constructs (*N* = 3). Scale bar 5 µm. The graph represents the percentage of fluorescence recovery of each aggregate over time compared to the baseline (i.e. 0 s) ± SD. Two-way ANOVA with Sidak’s post-test was performed comparing N999Vfs*40 and C975Vfs*73 KIF5A fluorescence recovery levels at each time point. *P < 0.05; ***P < 0.001.

On these bases, we compared the distribution of the two frameshift mutants and found that C975Vfs*73 KIF5A accumulated both in the cell body and within neurites into larger aggregates than those formed by the N999Vfs*40 mutant (Fig. 7A), in line with the presence of an additional low-solubility amino acid sequence in C975Vfs*73 KIF5A tail. Moreover, aggregates formed by the NEIMY-associated mutant sequestered WT KIF5A to a higher extent compared to N999Vfs*40 KIF5A ones (Fig. 7B), suggesting that C975Vfs*73 KIF5A might exert a more prominent dominant-negative effect on the WT protein. Additionally, as seen for N999Vfs*40 KIF5A, no co-distribution was observed between mitochondria and C975Vfs*73 KIF5A aggregates (Fig. 7C), hinting at a shared loss of function between ALS- and NEIMY-KIF5A mutants that would be consistent with reports implicating mitochondrial dysfunction in both phenotypes [13, 14, 26]. Notably, SQSTM1/p62 was co-immunoprecipitated by both N999Vfs*40 and C975Vfs*73 KIF5A (Supplementary Fig. 8A), but was found exclusively at the rim of NEIMY-KIF5A aggregates, while it was clearly observed within ALS-KIF5A ones (Supplementary Fig. 8B). Consistently, NP-40–soluble/insoluble protein extraction showed that both ALS- and NEIMY-KIF5A are characterised by poor detergent solubility; however, the former sequestered a larger amount of SQSTM1/p62 protein in the insoluble fraction in comparison to the latter (Fig. 7D). Finally, proteasomal blockage by MG132 induced C975Vfs*73 KIF5A accumulation, while autophagy inhibition did not significantly modify its protein levels, similarly to the N999Vfs*40 KIF5A mutant (Supplementary Fig. 8C). In line with this observation, both ALS- and NEIMY-KIF5A aggregates were found to be ubiquitinated and positive for the ubiquitin-binding protein BAG1 (Supplementary Fig. 8D).

A deeper characterisation of frameshift KIF5A inclusions also revealed features that are unique to NEIMY-KIF5A. Specifically, C975Vfs*73 KIF5A aggregates, but not those of N999Vfs*40 KIF5A, resulted positive for HDAC6 and G3BP1 (Fig. 7E, Supplementary Fig. 9A, and Supplementary Table 3), known to be implicated in stress granule formation [35]. Interestingly, live imaging analysis showed that both ALS- and NEIMY-KIF5A aggregates were motile and dynamic 24 h after transfection, but at 48 h, the largest C975Vfs*73 KIF5A aggregates had lost most of their motility differently from N999Vfs*40 KIF5A aggregates (Supplementary Fig. 9B). Consistently, while both ALS- and NEIMY-KIF5A aggregates recovered fluorescence following photobleaching 24 h after transfection (Supplementary Fig. 9C), only N999fs*40 KIF5A aggregates retained this ability at 48 h (Fig. 7F). This is in contrast with the extreme flexibility of stress granules and indicates that the initially dynamic C975fs*73 KIF5A-positive aggregates may mature into solid-like structures with time.

Together, these results suggest that most biochemical features characterising the ALS-linked N999Vfs*40 KIF5A mutant appear exacerbated for the NEIMY-related C975Vfs*73 KIF5A, providing a possible explanation for the higher severity of its associated neurodevelopmental phenotype compared to KIF5A-linked neurodegeneration.

## Discussion

Mutations targeting KIF5A give rise to distinct neurodegenerative and neurodevelopmental diseases depending on the affected protein domain, but the bases of this heterogeneity are unclear. Most mutations cluster in the motor domain and mainly associate with HSP/SPG10 [9] or CMT [10], while KIF5A tail variants associate with ALS [11, 12] and NEIMY [13, 14]. Variants in the stalk domain are less frequent and poorly characterised [36].

In this work, we investigated the biochemical behaviour of four disease-associated KIF5A mutants identified in a cohort of Italian patients, to discern the shared and unique pathogenic mechanisms underlying KIF5A-associated diseases. The clinical features of patients carrying the selected variants well illustrate the main aspects of phenotypic distinction and overlap characterising these disorders. A consistent phenotype is associated with motor domain variants (e.g. the recurrent p.R280C mutation), in which polyneuropathy with foot deformities and upper motor neuron signs are the main core features, often associated with ataxia and cognitive impairment. The occurrence of cognitive impairment in the two patients diagnosed with CMT5 further supports the notion that the clinical phenotypes associated with motor domain variants could be more appropriately described as complex HSP. Clinical heterogeneity is well illustrated by the cases of Patients 12 and 13. Patient 12, who carried the truncating variant p.R864* in the stalk domain, exhibited a mild neuropathic phenotype, while a previously reported patient with the same variant had a complex HSP [20]. Patient 13, carrying variant p.(N1006S), putatively belonging to the group of ALS-associated ΔExon27 frameshift tail mutations, had a late-onset and rapidly progressing ALS phenotype, unlike most patients with *KIF5A* tail mutations, who exhibit younger onset and slow course of the disease [11, 12].

The variety of clinical phenotypes caused by mutant KIF5A, along with its multifaceted functions, prompts the need to dissect and compare the molecular mechanisms of different variants in a homogeneous experimental setting. Previous studies have focused on single mutants [11, 15, 37], lacking the possibility of direct comparison we wanted to pursue in our study.

The first shared biochemical feature we identified between the KIF5A mutants under investigation is the peripheral localisation of the C-terminal mutants R864* and N999Vfs*40 KIF5A, consistent with the loss of KIF5A autoinhibitory function. Either mutations or deletion of the IAK motif generate constitutively active kinesin motors [3]. The IAK motif is absent in R864* KIF5A, possibly explaining its altered intracellular distribution. Differently, the loss of KIF5A autoinhibition observed for N999Vfs*40 KIF5A has been previously described [16–18], and it has been attributed to the replacement of 7 negatively charged amino acids with 9 positively charged ones in mutant KIF5A C-terminal domain; such change is predicted to hinder the stability of the head-tail association state required for kinesin autoinhibition [16]. At the functional level, autoinhibition loss causes the rapid removal of dimers containing mutant KIF5A from the pool of cytoplasmic motors available for anterograde transport, which in turn could cause imbalances in axonal trafficking, as previously suggested for ALS-KIF5A [16, 18]. Additionally, both R864* (by tail truncation) and N999Vfs*40 KIF5A (by translational frameshift of its cargo/adaptor-binding region) may lose their ability to interact with KIF5A-specific substrates. However, the adaptor binding sites on the stalk domain are not completely abolished by the R864* truncating mutation (e.g. kinesin light chain binding involves amino acids 770-811 [38]), thus its interaction with specific cargoes could be partially retained. Similarly, the ability of N999Vfs*40 KIF5A to transport cargo is debated. While Baron and colleagues [16] reported increased speed and percentage of moving mitochondria in ALS-KIF5A- compared to WT KIF5A-overexpressing primary motor neurons, Pant et al. [18] did not observe co-distribution between ALS-KIF5A inclusions and mitochondria in overexpressing primary cortical neurons, in line with our data. The absence of co-distribution between ALS-KIF5A and mitochondria does not necessarily reflect the inability of KIF5A motors to interact with their cargo, since this interaction may be lost only upon ALS-KIF5A oligomerisation and aggregation. While distal accumulation of mitochondria (or other KIF5A cargoes) may be promoted by soluble ALS-KIF5A motors or WT-mutant KIF5A heterodimers, the inability to recycle aggregated ALS-KIF5A to the cytoplasm might also cause an opposite effect of enhanced retrograde transport, introducing additional imbalances in axonal trafficking. Moreover, this effect may be exacerbated by WT KIF5A sequestration into ALS-KIF5A aggregates and might not be compensated by other kinesin motors. Alterations in transport equilibrium may also occur with the R864* KIF5A mutant, which could promote the fast dismissal of the WT protein from the cytoplasm via heterodimerisation, even if additional studies are required to better elucidate these aspects.

A second shared behaviour we observed is between the SPG10-related R17Q KIF5A mutant and N999Vfs*40 KIF5A, that both display a shorter turnover compared to the WT protein, with which they dimerise. Thus, the fast turnover of R17Q and N999Vfs*40 KIF5A may affect the clearance of both their homodimers and WT-mutant KIF5A heterodimers, restricting the pool of motors available for axonal transport. Concerning the R17Q KIF5A mutant, the alteration of its ATP-binding site predicted in silico may tamper with its ability to fuel transport, and also destabilise the protein, causing its premature degradation, in line with the reported loss of function for SPG10-related *KIF5A* variants [15]. Regarding the accelerated turnover of N999Vfs*40 KIF5A, this is in line with its lower levels compared to WT KIF5A in iPSC-derived motor neurons harbouring ALS-KIF5A mutations [16, 39] and in ALS-KIF5A animal models [27, 40].

The other SPG10-related mutant, R280C KIF5A, also shares similarities with other KIF5A variants in our study. The R280C substitution targets the microtubule-binding site of the KIF5A head, reducing its affinity for microtubule tracks without having a dominant-negative effect on the WT protein. Nonetheless, the R280C mutant competes with WT KIF5A for cargo binding, sequestering substrates to transport-incompetent KIF5A motors [15], a mechanism that could be shared with R17Q KIF5A, which has an inactive ATP-binding site but an intact tail. Notably, we found that R280C KIF5A forms small perinuclear puncta, potentially due to its inability to interact with microtubules, and competes with Ub-R-YFP for proteasomal degradation, that are features also described for N999Vfs*40 KIF5A.

Finally, we found that the NEIMY-linked C975Vfs*73 KIF5A mutant shares most of the abnormal behaviours observed for N999Vfs*40 KIF5A, including loss of autoinhibition and distal distribution, aggregation propensity, negative dominance on WT KIF5A, and lack of co-distribution between aggregates and mitochondria. Despite all these similarities, the N999Vfs*40 and C975Vfs*73 KIF5A mutants lead to strikingly different neurological conditions, respectively an adult-onset form of motor neuron degeneration and a complex and very severe early-onset neurodevelopmental phenotype. Such difference might be related to the higher aggregation propensity and sequestration potential characterising NEIMY-KIF5A compared to ALS-KIF5A. Based on our observations, it is conceavable that NEIMY-KIF5A inclusions may form similarly to stress granules and then evolve into solid-like aggregates, loosing their dynamic status and the ability to undergo liquid-liquid phase partitioning typical of ribonucleoprotein condensates [35]. Protein and/or mRNA sequestration into these solid-like structures could further exacerbate NEIMY-KIF5A toxicity through both gain- and loss-of-function mechanisms.

The clinical symptoms affecting NEIMY patients are similar regardless of the underpinning mutation [13, 14], and they have been previously proposed to derive from mitochondrial dysfunction and altered excitatory/inhibitory equilibrium [13, 14, 41], defects that are in line with impaired KIF5A-dependent trafficking of mitochondria [6, 7] and GABA_A_ receptor subunits [42]. Interestingly, post-natal neuron-specific *Kif5a* depletion in conditional knock-out mice results in an epileptic phenotype [42] reminiscent of NEIMY patients, further supporting the hypothesis of a KIF5A loss of function component in this neurodevelopmental disease. Notably, accumulation of synaptic vesicles leading to alterations in synaptic transmission was recently observed in ALS-KIF5A *Drosophila* larvae [27], suggesting that this loss of function mechanism, too, could be shared between ALS and NEIMY. Altogether, our data on N999Vfs*40 and C975Vfs*73 KIF5A indicate that a combination of a loss of function in axonal transport and a toxic gain of function due to aggregation and WT KIF5A sequestration may underpin both NEIMY and KIF5A-linked ALS.

In conclusion, we performed a comprehensive functional study of a panel of *KIF5A* variants representative of the different *KIF5A*-related disorders. We demonstrated the pathogenicity of the novel p.R17Q variant, altered at the level of the ATP-binding site of the motor domain, and of the p.R864* variant, which is both one of the few mutations in the stalk domain and one of the very rare truncations in KIF5A. We also provided evidence for a combined gain/loss of function pathomechanism unexpectedly shared by two frameshift mutants associated with ALS and NEIMY, that would bridge the dichotomy between *KIF5A*-linked neurodevelopmental and neurodegenerative conditions.

## Materials and methods

### Genetic studies

Patients were recruited by the Units of Medical Genetics and Neurogenetics, Rare Neurological Diseases, and Neurodevelopmental Disorders at the Istituto Neurologico Carlo Besta (Milan, Italy) and the Neurology Unit at the Azienda Ospedaliero-Universitaria di Modena (Modena, Italy).

Genetic analysis of the *KIF5A* gene was performed by Sanger sequencing or by targeted next generation sequencing approaches (single-gene amplicon-deep sequencing or disease gene panels) on Illumina MiSeq or NextSeq550 apparatuses (Illumina, Inc., San Diego, CA, USA). Novel variants were classified according to the guidelines of the ACMG [19]. In silico prediction of the exon 27 variant was evaluated by Alternative Splice Site Predictor [43], NNSplice [44], SpliceAI [45], and dbscSNV Ada/RF [46] tools. All variants are described in relation to the reference sequence NM_004984.4 and according to current Human Genome Variation Society recommendations for sequence variant nomenclature (https://varnomen.hgvs.org/; Mutalyzer 3: https://mutalyzer.nl).

### Chemicals and antibodies

CHX (C6255), MG132 (C2211) and NH_4_Cl (254134) were purchased from Sigma-Aldrich (Saint Louis, MI, USA). 10 μg/ml CHX was used to treat cells for 1-2-4-6 h to block protein synthesis and evaluate KIF5A stability. 10 µM MG132 was used to treat cells for 16 h to block the 26S proteasome. 20 mM NH_4_Cl was used to treat cells for 16 h to inhibit autophagy.

All antibodies used in the present work are listed in Supplementary Table 4.

### Plasmids

pKIF5A plasmids for the transient overexpression of human WT or mutant (R17Q, R280C, R864*, N999Vfs*40, C975Vfs*73) KIF5A were generated by GenScript Biotech (Piscataway, NJ, USA) by cloning the cDNA sequences of interest into the backbone of the empty vector (EV) pcDNA3.1. pFLAG-KIF5A and pGFP-KIF5A were cloned starting from the pKIF5A constructs. A complete list of the plasmids used in this work is reported in Supplementary Table 5.

### Cell lines and transfection

The human neuroblastoma SH-SY5Y cell line was obtained from the American Type Culture Collection (ATCC, Rockville, MD, USA CRL-2266;). SH-SY5Y cells were cultured at 37 °C in 5% CO_2_ in DMEM High Glucose (Euroclone S.p.A., Pero, Italy; ECB7501L) supplemented with 10% heat-inactivated foetal bovine serum (FBS; Sigma-Aldrich, Saint Louis, MI, USA; F7524), 1 mM l-glutamine (Euroclone S.p.A., Pero, Italy; ECB3004D), and antibiotics (SERVA Electrophoresis GmbH, Heidelberg, Germany; penicillin, 31749.04; streptomycin, 35500.01).

The murine motor neuron-like NSC-34 cell line was provided by Dr. Neil R. Cashman (University of British Columbia, Canada). NSC-34 cells were cultured at 37 °C in 5% CO_2_ in DMEM High Glucose supplemented with 5% FBS, 1 mM l-glutamine and antibiotics.

The SH-SY5Y neuroblastoma cell line was chosen to investigate mutant KIF5A biochemical behaviour because of its human origin. The murine NSC-34 cell line was used to analyse mutant KIF5A distribution and motility in a bona fide motoneuronal model not requiring differentiation.

SH-SY5Y and NSC-34 cells were transfected with Lipofectamine™ 3000 Transfection Reagent (Thermo Fisher Scientific Inc., Waltham, MA, USA; L3000015) diluted in Opti-MEM™ (Thermo Fisher Scientific Inc., Waltham, MA, USA; 31985070) 24 h after seeding, according to the manufacturer’s instructions. The next day cells were directly processed (SH-SY5Y cells, co-immunoprecipitation or CHX assay), or the medium was changed to limit Lipofectamine™ 3000 toxicity.

### Fluorescence microscopy and immunofluorescence

NSC-34 cells were seeded in 24-well plates onto 13-mm coverslips at 35,000 cells/well and transfected as previously described. 48 h after transfection, cells were fixed onto coverslips in a 1:1 solution of 4% paraformaldehyde and 4% sucrose in 0.2 M phosphate buffer (0.06 M KH_2_PO_4_, 0.31 M Na_2_HPO_4_; pH 7.4) for 25 min at 37 °C. For fluorescence microscopy analyses, nuclei were stained with 0.02% DAPI in PBS and coverslips were mounted with Mowiol® 4-88 Reagent (Sigma-Aldrich, Saint Louis, MI, USA; 475904). For immunofluorescence analyses, cells were incubated in PBS added with 0.1% Triton X-100, 1% BSA (Sigma-Aldrich, Saint Louis, MI, USA; X100 and A7030), and 10% FBS for 45 min at room temperature for permeabilisation and blocking of nonspecific sites. Subsequently, cells were incubated with the required primary and then secondary antibodies diluted in PBS added with 0.1% BSA as reported in Supplementary Table 4. Finally, nuclei were stained with DAPI, and coverslips were mounted as described. Images were acquired with an LSM 900 confocal microscope (Carl Zeiss Microscopy, Germany) and were processed using ImageJ/Fiji (version 2.9.0).

### Western blot

SH-SY5Y cells were seeded in 12-well plates at 150,000 cells/well and transfected as previously described. At the time of harvesting, cells were collected in their own medium and centrifuged at 100×*g* for 5 min at 4 °C to remove the supernatant. Total proteins were then extracted and protein concentration was determined through bicinchoninic acid assay with the QPRO BCA Kit Standard (Cyanagen Srl, Bologna, Italy; PRTD1), following the manufacturer’s instructions. Subsequently, 15–20 μg total proteins were incubated in sample buffer (250 mM Tris, 40% glycerol, 8% SDS, 0.1% bromophenol blue) at 100 °C for 5 min for denaturation and loaded on 10–15% SDS-polyacrylamide gels. Separated proteins were then transferred to 0.45-µm nitrocellulose membranes (Euroclone S.p.A., Pero, Italy; GE10600002) using a Trans-Blot® Turbo^TM^ Transfer System (Bio-Rad Laboratories, Hercules, CA, USA). For the immunochemical detection of proteins, membranes were initially incubated in 5% non-fat dried milk diluted in TBS-T (20 mM Tris-HCl pH 7.5, 0.5 M NaCl, 0.05% Tween 20; pH 7.6) to block nonspecific sites and then probed using the antibodies reported in Supplementary Table 4. Immunoreactivity was detected using Westar chemiluminescent reagents (Cyanagen Srl, Bologna, Italy). Images were acquired using a ChemiDoc^TM^ XRS+ System (Bio-Rad Laboratories, Hercules, CA, USA), and optical density was analysed with Image Lab^TM^ Software (Bio-Rad Laboratories, Hercules, CA, USA; version 6.0.1).

To analyse KIF5A solubility, SH-SY5Y cells were harvested 48 h after transfection as described, resuspended in NP-40 lysis buffer (150 mM NaCl, 20 mM Tris base, 0.05% NP-40, 1.5 mM MgCl_2_, 3% glycerol; pH 7.4) added with 1 mM DTT (Sigma-Aldrich, Saint Louis, MI, USA; 3483-12-3) and Protease Inhibitor Cocktail (Sigma-Aldrich, Saint Louis, MI, USA; P8340), and passed 10 times through a syringe needle for lysis. Then, 20 μg total proteins were diluted in 15 μl NP-40 lysis buffer and were centrifuged at 16,100×*g* for 15 min at 4 °C. NP-40–soluble supernatants were collected, while NP-40–insoluble pellets were resuspended in the same volume of NP-40 lysis buffer (without protease inhibitor and DTT) and sonicated. Both NP-40–soluble and NP-40-insoluble fractions were then analysed.

To compare WT and mutant KIF5A turnover, 24 h after transfection SH-SY5Y cells were incubated with fresh medium added with CHX and collected at the previously indicated time points, except for control samples which were immediately harvested. After the last collection, cells were resuspended in PBS added with protease inhibitor and lysed through slight sonication to extract proteins.

In all other cases, SH-SY5Y cells were harvested 48 h after transfection and processed to extract proteins in PBS added with protease inhibitor as described.

### RNA extraction from cells and RT-qPCR

SH-SY5Y cells were seeded in 12-well plates at 150,000 cells/well and transfected as previously described. 48 h after transfection, total RNA was extracted from cells using TRI Reagent® (Sigma-Aldrich, Saint Louis, MI, USA; T9424) and 1-bromo-3-chloropropane (Sigma-Aldrich, Saint Louis, MI, USA; B9673), following the manufacturer’s instructions. RNA quantification was performed using a NanoDrop 2000 (Thermo Fisher Scientific Inc., Waltham, MA, USA), after which 1 μg/sample RNA was treated with DNase I (Sigma-Aldrich, Saint Louis, MI, USA; AMPD1) and reverse transcribed to cDNA with the High-Capacity cDNA Reverse Transcription Kit (Thermo Fisher Scientific Inc., Waltham, MA, USA; 4368814). Subsequently, qPCR was performed using the iTaq SYBR Green Supermix (Bio-Rad Laboratories, Hercules, CA, USA; 1725124) in a total volume of 10 μl with 500 nmol primers. A CFX96 Real-Time System (Bio-Rad Laboratories, Hercules, CA, USA) was used according to the following cycling conditions: 94 °C for 10 min, 94 °C for 15 s (40 cycles), 6 °C for 1 min. Data were expressed as C_t_ values and analysed with CFX Manager™ Software (Bio-Rad Laboratories, Hercules, CA, USA; version 3.1). Primers for qPCR were synthesised by Eurofins MWG-Biotech (Ebersberg, Germany) with the following sequences: *hKIF5A* 5′–GGAGAACATGGAAACGGAGCA–3′ (forward), 5′–TATTCTTTGCCTCGTCCAGCAC–3′ (reverse); *hGAPDH* 5′–GAAGGTGAAGGTCGGAGTC–3′ (forward), 5′ –TTGATGGCAACAATATCCACTT–3′ (reverse).

### Co-immunoprecipitation

SH-SY5Y cells were seeded in 6-well plates at 300,000 cells/well and transfected as previously described. The next day, cells were harvested in their medium pooling three wells per experimental condition and centrifuged at 100×*g* for 5 min at 4 °C. Pellets were resuspended in RIPA buffer (150 mM NaCl, 0.5% Na-deoxycholate, 100 µM Na-orthovanadate, 50 mM NaF, 50 mM Tris-HCl pH 7.7, 10 mM EDTA pH 8, 0.08% SDS, 0.8% Triton X-100) added with cOmplete Protease Inhibitor Cocktail (Sigma-Aldrich, Saint Louis, MI, USA; 4693116001), incubated on ice for 20 min, and centrifuged at 16,000×*g* for 15 min at 4 °C for clearing. 100 μl/sample SureBeads^TM^ Protein G Magnetic Beads (Bio-Rad Laboratories, Hercules, CA, USA; 1614023) were conjugated to 2 μg/sample mouse monoclonal anti-FLAG antibody (Sigma-Aldrich, Saint Louis, MI, USA; F1804) diluted in PBS-T (PBS, 0.1% Tween 20) for 10 min at room temperature and washed in PBS-T. Antibody–conjugated beads were then incubated with 250 μg/sample RIPA-soluble protein extracts for 1 h at 4 °C. After washing in PBS-T, immunoprecipitated proteins were eluted from the beads with Laemmli Sample Buffer (Bio-Rad Laboratories, Hercules, CA, USA; 1610737) added with 5% β-mercaptoethanol by incubating samples for 10 min at 70 °C. Immunoprecipitation, input, and output samples were then loaded on 7.5% gels and analysed through a western blot.

### Proteasome activity assay

SH-SY5Y cells were seeded in 6-well plates at 300,000 cells/well and transfected as previously described. Un-transfected samples were treated with 1 μM MG132 for 16 h to block the 26S proteasome as control. 48 h after transfection, cells were harvested in their medium, centrifuged at 600×*g* for 5 min, and washed three times in PBS. Pellets were then homogenised in PBS added with 0.5% NP-40 and centrifuged at 1300×*g* for 15 min. Supernatants were subsequently collected and protein concentration was quantified as described. Reaction mixtures were prepared by diluting 50 μg total proteins in 50 mM HEPES-KOH pH 8.0 and added with 5 mM EGTA and 5 mM ATP to a final volume of 500 μl. 50 nM AMC-conjugated substrates were added to the reaction mixtures to quantify the chymotryptic-like (Sigma-Aldrich, Saint Louis, MI, USA; *N*-Suc-LLVY-AMC, S6510) and the caspase-like (Sigma-Aldrich, Saint Louis, MI, USA; Z-LLE-AMC, C0483) activities of the 26S proteasome. Finally, samples were incubated for 45 min at 37 °C and fluorescence was measured at 340 nm excitation and 460 nm emission wavelengths using an Enspire® Multimode Plate Reader (PerkinElmer, Inc., Waltham, MA, USA).

### Protein solubility predictions

CamSol (http://www-vendruscolo.ch.cam.ac.uk/camsolmethod.html; accessed on November 20, 2022) was used to predict the intrinsic solubility profile of WT, N999Vfs*40, and C975Vfs*73 KIF5A C-terminal tails with the CamSol Intrinsic method [34]. The following sequences were used as input:

YFANSCTSSGATSSGGPLASYQKANMDNGNATDINDNRSDLPCGYEAEDQAKLFPLHQETAAS for WT KIF5A (amino acids 970–1032);

YFANSCTSSGATSSGGPLASYQKANMDNGVTCRVAMRLRTRPSFSLSTKRQQPANLPHPRLHTCTFSF for N999Vfs*40 KIF5A (amino acids 970–1037);

YFANSVPAVDPHLLAAPWLPTRRPTWTMDMPQISMTIGVTCRVAMRLRTRPSFSLSTKRQQPANLPHPRLHTCTFSF for C975Vfs*73 KIF5A (amino acids 970–1046).

### Live imaging

NSC-34 cells were seeded in 35-mm glass bottom dishes at 50,000 cells/well and transfected as previously described. 24 h or 48 h after transfection, cells were imaged in their medium added with 20 mM HEPES pH 7.4 at 37 °C and 5% CO_2_ with an LSM 900 confocal microscope using a 63× oil-immersion lens. Live imaging sequences were acquired at 1 frame every 1.5–2 s for up to 300 s. For fluorescence recovery after photobleaching, one pre-bleach image preceded photobleaching with a 5 mW 488 nm laser at 100% for 3 s. Images were processed using ImageJ/Fiji (version 2.9.0).

### Statistics

Unpaired two-sided Student’s *t*-test and one- or two-way ANOVA tests followed by Fisher’s LSD or Sidak’s post-tests were applied, according to figure captions. Gaussian distribution was assumed in all cases and equal variance was confirmed by Brown–Forsythe test. *P* < 0.05 was considered significant. All analyses were performed using GraphPad PRISM (version 8.0.2). For the CHX chase assay (Fig. 3D), images in which bands corresponding to WT or mutant KIF5A baseline (i.e. 0 h) displayed comparable optical densities were chosen for data analysis in order to set a virtually identical reference value between the tested conditions, even if the images were acquired at different exposure times. For each experimental condition, mean normalised optical densities of the reference bands were set at 100 and all other mean values were plotted as a percentage of the reference values.

## Supplementary information

Supplementary figures

Supplementary Table 1 WORD Format

Supplementary Table 2 WORD Format

Supplementary Table 3 WORD Format

Supplementary Table 4 WORD Format

Supplementary Table 5 WORD Format

Supplementary immunofluorescence images

Unedited blot Images

## Data Availability

Data supporting the findings of this study are available from the corresponding authors upon request. Anonymised data from this study are available at https://zenodo.org/communities/besta and will be shared by request from any qualified investigator.

## References

[1] Hirokawa N, Noda Y, Tanaka Y, Niwa S. Kinesin superfamily motor proteins and intracellular transport. Nat Rev Mol Cell Biol. 2009;10:682–96. 19773780 10.1038/nrm2774

[2] Miki H, Okada Y, Hirokawa N. Analysis of the kinesin superfamily: insights into structure and function. Trends Cell Biol. 2005;15:467–76. 16084724 10.1016/j.tcb.2005.07.006

[3] Kaan HYK, Hackney DD, Frank K. The structure of the Kinesin-1 motor-tail complex reveals the mechanism of autoinhibition. Science. 2011;333:883–5. 21836017 10.1126/science.1204824 PMC3339660

[4] Xia C-H, Roberts EA, Her L-S, Liu X, Williams DS, Cleveland DW, et al. Abnormal neurofilament transport caused by targeted disruption of neuronal kinesin heavy chain KIF5A. J Cell Biol. 2003;161:55–66. 12682084 10.1083/jcb.200301026 PMC2172877

[5] Kanai Y, Dohmae N, Hirokawa N. Kinesin transports RNA: isolation and characterization of an RNA-transporting granule. Neuron. 2004;43:513–25. 15312650 10.1016/j.neuron.2004.07.022

[6] Karle KN, Möckel D, Reid E, Schöls L. Axonal transport deficit in a KIF5A(-/-) mouse model. Neurogenetics. 2012;13:169–79. 22466687 10.1007/s10048-012-0324-y PMC3332386

[7] Campbell PD, Shen K, Sapio MR, Glenn TD, Talbot WS, Marlow FL. Unique function of Kinesin Kif5A in localization of mitochondria in axons. J Neurosci. 2014;34:14717–32. 25355224 10.1523/JNEUROSCI.2770-14.2014 PMC4212069

[8] Liu M, Pi H, Xi Y, Wang L, Tian L, Chen M, et al. KIF5A-dependent axonal transport deficiency disrupts autophagic flux in trimethyltin chloride-induced neurotoxicity. Autophagy. 2021;17:903–24. 32160081 10.1080/15548627.2020.1739444 PMC8078766

[9] Reid E, Kloos M, Ashley-Koch A, Hughes L, Bevan S, Svenson IK, et al. A kinesin heavy chain (KIF5A) mutation in hereditary spastic paraplegia (SPG10). Am J Hum Genet. 2002;71:1189–94. 12355402 10.1086/344210 PMC385095

[10] Crimella C, Baschirotto C, Arnoldi A, Tonelli A, Tenderini E, Airoldi G, et al. Mutations in the motor and stalk domains of KIF5A in spastic paraplegia type 10 and in axonal Charcot-Marie-Tooth type 2. Clin Genet. 2012;82:157–64. 21623771 10.1111/j.1399-0004.2011.01717.x

[11] Nicolas A, Kenna KP, Renton AE, Ticozzi N, Faghri F, Chia R, et al. Genome-wide analyses identify KIF5A as a novel ALS gene. Neuron. 2018;97:1268–1283.e6. 10.1016/j.neuron.2018.02.027 PMC5867896 29566793

[12] Brenner D, Yilmaz R, Müller K, Grehl T, Petri S, Meyer T, et al. Hot-spot KIF5A mutations cause familial ALS. Brain. 2018;141:688–97. 29342275 10.1093/brain/awx370 PMC5837483

[13] Duis J, Dean S, Applegate C, Harper A, Xiao R, He W, et al. KIF5A mutations cause an infantile onset phenotype including severe myoclonus with evidence of mitochondrial dysfunction. Ann Neurol. 2016;80:633–7. 27463701 10.1002/ana.24744 PMC5042851

[14] Rydzanicz M, Jagła M, Kosinska J, Tomasik T, Sobczak A, Pollak A, et al. KIF5A de novo mutation associated with myoclonic seizures and neonatal onset progressive leukoencephalopathy. Clin Genet. 2017;91:769–73. 27414745 10.1111/cge.12831

[15] Ebbing B, Mann K, Starosta A, Jaud J, Schöls L, Schüle R, et al. Effect of spastic paraplegia mutations in KIF5A kinesin on transport activity. Hum Mol Genet. 2008;17:1245–52. 18203753 10.1093/hmg/ddn014

[16] Baron DM, Fenton AR, Saez-Atienzar S, Giampetruzzi A, Sreeram A. Shankaracharya et al. ALS-associated KIF5A mutations abolish autoinhibition resulting in a toxic gain of function. Cell Rep. 2022;39:110598. 35385738 10.1016/j.celrep.2022.110598 PMC9134378

[17] Nakano J, Chiba K, Niwa S. An ALS‐associated KIF5A mutant forms oligomers and aggregates and induces neuronal toxicity. Genes Cells. 2022;27:421–35. 35430760 10.1111/gtc.12936 PMC9322661

[18] Pant DC, Parameswaran J, Rao L, Loss I, Chilukuri G, Parlato R, et al. ALS‐linked KIF5A ΔExon27 mutant causes neuronal toxicity through gain‐of‐function. EMBO Rep. 2022;23:e54234. 10.15252/embr.202154234 PMC9346498 35735139

[19] Richards S, Aziz N, Bale S, Bick D, Das S, Gastier-Foster J, et al. Standards and guidelines for the interpretation of sequence variants: a joint consensus recommendation of the American College of Medical Genetics and Genomics and the Association for Molecular Pathology. Genet Med. 2015;17:405–24. 25741868 10.1038/gim.2015.30 PMC4544753

[20] Lynch DS, Koutsis G, Tucci A, Panas M, Baklou M, Breza M, et al. Hereditary spastic paraplegia in Greece: characterisation of a previously unexplored population using next-generation sequencing. Eur J Hum Genet. 2016;24:857–63. 26374131 10.1038/ejhg.2015.200 PMC4688955

[21] Santangelo S, Bossolasco P, Magri S, Colombrita C, Invernizzi S, Gellera C, et al. Generation of an iPSC line from a patient with spastic paraplegia type 10 carrying a novel mutation in KIF5A gene. Stem Cell Res. 2023;66:103008. 36565680 10.1016/j.scr.2022.103008

[22] Ioannidis NM, Rothstein JH, Pejaver V, Middha S, McDonnell SK, Baheti S, et al. REVEL: an ensemble method for predicting the pathogenicity of rare missense variants. Am J Hum Genet. 2016;99:877–85. 27666373 10.1016/j.ajhg.2016.08.016 PMC5065685

[23] Rentzsch P, Witten D, Cooper GM, Shendure J, Kircher M. CADD: predicting the deleteriousness of variants throughout the human genome. Nucleic Acids Res. 2019;47:D886–94. 30371827 10.1093/nar/gky1016 PMC6323892

[24] Cedarbaum JM, Stambler N, Malta E, Fuller C, Hilt D, Thurmond B, et al. The ALSFRS-R: a revised ALS functional rating scale that incorporates assessments of respiratory function. J Neurol Sci. 1999;169:13–21. 10540002 10.1016/s0022-510x(99)00210-5

[25] DeBoer SR, You Y, Szodorai A, Kaminska A, Pigino G, Nwabuisi E, et al. Conventional kinesin holoenzymes are composed of heavy and light chain homodimers. Biochemistry. 2008;47:4535–43. 18361505 10.1021/bi702445j PMC2644488

[26] Pareyson D, Saveri P, Sagnelli A, Piscosquito G. Mitochondrial dynamics and inherited peripheral nerve diseases. Neurosci Lett. 2015;596:66–77. 25847151 10.1016/j.neulet.2015.04.001

[27] Soustelle L, Aimond F, Andrés CL, Brugioti V, Raoul C, Layalle S. ALS-associated KIF5A mutation causes locomotor deficits associated with cytoplasmic inclusions, alterations of neuromuscular junctions and motor neuron loss. J Neurosci. 2023;43:8058–72. 10.1523/JNEUROSCI.0562-23.2023 PMC10669773 37748861

[28] Liu X, Klionsky DJ. Regulation of autophagic lysosome reformation by kinesin 1, clathrin and phosphatidylinositol-4,5-bisphosphate. Autophagy. 2018;14:1–2. 28980869 10.1080/15548627.2017.1386821 PMC5846503

[29] Toupenet Marchesi L, Leblanc M, Stevanin G. Current knowledge of endolysosomal and autophagy defects in hereditary spastic paraplegia. Cells 2021;10:1678. 10.3390/cells10071678 PMC8307360 34359848

[30] Cozzi M, Ferrari V. Autophagy dysfunction in ALS: from transport to protein degradation. J Mol Neurosci. 2022;72:1456–81. 35708843 10.1007/s12031-022-02029-3 PMC9293831

[31] Pankiv S, Clausen TH, Lamark T, Brech A, Bruun J-A, Outzen H, et al. p62/SQSTM1 binds directly to Atg8/LC3 to facilitate degradation of ubiquitinated protein aggregates by autophagy. J Biol Chem. 2007;282:24131–45. 17580304 10.1074/jbc.M702824200

[32] Myeku N, Figueiredo-Pereira ME. Dynamics of the degradation of ubiquitinated proteins by proteasomes and autophagy. J Biol Chem. 2011;286:22426–40. 21536669 10.1074/jbc.M110.149252 PMC3121389

[33] Menéndez-Benito V, Verhoef LGGC, Masucci MG, Dantuma NP. Endoplasmic reticulum stress compromises the ubiquitin–proteasome system. Hum Mol Genet. 2005;14:2787–99. 16103128 10.1093/hmg/ddi312

[34] Sormanni P, Vendruscolo M. Protein solubility predictions using the CamSol method in the study of protein homeostasis. Cold Spring Harb. Perspect. Biol. 2019;11:a033845. 10.1101/cshperspect.a033845 PMC6886446 30833455

[35] Riggs CL, Kedersha N, Ivanov P, Anderson P. Mammalian stress granules and P bodies at a glance. J Cell Sci. 2020;133:jcs242487. 10.1242/jcs.242487 PMC10679417 32873715

[36] de Boer EMJ, van Rheenen W, Goedee HS, Kamsteeg E-J, Brilstra EH, Veldink JH, et al. Genotype-phenotype correlations of KIF5A stalk domain variants. Amyotroph Lateral Scler Frontotemporal Degener. 2021;22:561–70. 33829936 10.1080/21678421.2021.1907412

[37] Dutta M, Diehl MR, Onuchic JN, Jana B. Structural consequences of hereditary spastic paraplegia disease-related mutations in kinesin. Proc Natl Acad Sci USA. 2018;115:E10822–29. 30366951 10.1073/pnas.1810622115 PMC6243270

[38] Diefenbach RJ, Mackay JP, Armati PJ, Cunningham AL. The C-terminal region of the stalk domain of ubiquitous human kinesin heavy chain contains the binding site for kinesin light chain. Biochemistry. 1998;37:16663–70. 9843434 10.1021/bi981163r

[39] Pino MG, Rich KA, Hall NJ, Jones ML, Fox A, Musier-Forsyth K, et al. Heterogeneous splicing patterns resulting from KIF5A variants associated with amyotrophic lateral sclerosis. Hum Mol Genet. 2023;32:3166–80. 10.1093/hmg/ddad134 37593923

[40] Rich KA, Pino MG, Yalvac ME, Fox A, Harris H, Balch MHH, et al. Impaired motor unit recovery and maintenance in a knock-in mouse model of ALS-associated Kif5a variant. Neurobiol Dis. 2023;182:106148. 37164288 10.1016/j.nbd.2023.106148 PMC10874102

[41] Fukuoka M, Okazaki S, Kim K, Nukui M, Inoue T, Kuki I, et al. Preliminary report for epilepsia open: a case of West syndrome with severe global developmental delay and confirmed KIF5A gene variant. Epilepsia Open. 2021;6:230–4. 33681666 10.1002/epi4.12431 PMC7918309

[42] Nakajima K, Yin X, Takei Y, Seog D-H, Homma N, Hirokawa N. Molecular motor KIF5A is essential for GABA(A) receptor transport, and KIF5A deletion causes epilepsy. Neuron. 2012;76:945–61. 23217743 10.1016/j.neuron.2012.10.012

[43] Wang M, Marín A. Characterization and prediction of alternative splice sites. Gene. 2006;366:219–27. 16226402 10.1016/j.gene.2005.07.015

[44] Reese MG, Eeckman FH, Kulp D, Haussler D. Improved splice site detection in Genie. J Comput. Biol. 1997;4:311–23. 9278062 10.1089/cmb.1997.4.311

[45] Jaganathan K, Kyriazopoulou Panagiotopoulou S, McRae JF, Darbandi SF, Knowles D, Li YI, et al. Predicting splicing from primary sequence with deep learning. Cell. 2019;176:535–548.e24. 30661751 10.1016/j.cell.2018.12.015

[46] Jian X, Boerwinkle E, Liu X. In silico prediction of splice-altering single nucleotide variants in the human genome. Nucleic Acids Res. 2014;42:13534–44. 25416802 10.1093/nar/gku1206 PMC4267638

