# Supplementary material for: Altered molecular and cellular mechanisms in KIF5A-associated neurodegenerative or neurodevelopmental disorders
Source: Cell Death Dis. 2024 Sep 27;15(9):692. doi: 10.1038/s41419-024-07096-5 (PMC11437142; doi:10.1038/s41419-024-07096-5)
Supplement: Supplementary file 2 — Supplementary Table 1 WORD Format [file 41419_2024_7096_MOESM2_ESM.docx]

**Supplementary Table 1 List of pathogenic *KIF5A* variants (updated: February 28^th^, 2024)**

| **Gene** | **Protein** | **Domain** | **Phenotype** | **Reference** | **Note** |
| --- | --- | --- | --- | --- | --- |
| c.50G>A | p.R17Q | Motor | SPG10 | This study |  |
| c.130-943_c.1717-533del | p.G44_L572del | Motor | CMT | Pyromali 2021^1^ |  |
| c.188A>G | p.Y63C | Motor | SPG10 | Goizet 2009^2^ |  |
| c.217G>A | p.D73N | Motor | SPG10 | Schüle 2008^3^ | abnormal splicing prediction |
| c.259C>G | p.Q87E | Motor | SPG10 with cerebellar ataxia | Qiu 2018^4^ |  |
| c.332G>C | p.R111P | Motor | CMT | Bacquet 2018^5^ |  |
| c.340C>T | p.R114* | Motor | ALS | Grassano 2022^6^ |  |
| c.395A>G | p.K132R | Motor | SPG10, CMT | Bacquet 2018^5^ Méreaux 2022^7^ |  |
| c.396G>A | p.K132K | Motor | SPG10 | van de Warrenburg 2016^8^ | abnormal splicing prediction |
| c.416A>G | p.Y139C | Motor | SPG10 | Méreaux 2022^7^ |  |
| c.484C>T | p.R162W | Motor | SPG10 | Carosi 2015^9^ Kaji 2016^10^ Elert-Dobkowska 2019^11^ |  |
| c.485G>C | p.R162P | Motor | SPG10 | Oliveira 2021^12^ |  |
| c.572G>A | p.R191H | Motor | SPG10 | Morais 2017^13^ Méreaux 2022^7^ |  |
| c.580G>C | p.A194P | Motor | SPG10 | Collongues 2013^14^ |  |
| c.587C>A | p.T196N | Motor | CMT | Cortese 2020^15^ |  |
| c.593T>C | p.M198T | Motor | SPG10 | Goizet 2009^2^ |  |
| c.604A>G | p.S202G | Motor | SPG10 | Méreaux 2022^7^ |  |
| c.605G>A | p.S202N | Motor | SPG10 | Crimella 2012^16^ Méreaux 2022^7^ |  |
| c.608C>G | p.S203C | Motor | SPG10 | Musumeci 2011^17^ |  |
| c.610C>T | p.R204W | Motor | SPG10 (+/- cerebellar ataxia), CMT | Tessa 2008^18^ Liu 2014^19^ Morais 2017^13^ Nam 2018^20^  Cuchanski 2018^21^  He 2020^22^  Méreaux 2022^7^ |  |
| c.611G>A | p.R204Q | Motor | SPG10 | Goizet 2009^2^ Crimella 2012^16^ Neveling 2013^23^ Liu 2014^19^ Orsucci 2014^24^ Jerath 2015^25^  Lee 2020^26^  Méreaux 2022^7^ |  |
| c.687T>A | p.Y229* | Motor | SPG10 | Smedley 2021^27^ |  |
| c.691G>T | p.V231L | Motor | SPG10 | Crimella 2012^16^ |  |
| c.694G>A | p.D232N | Motor | CMT | Liu 2014^19^ Dellatte 2023^28^ |  |
| c.704G>A | p.G235E | Motor | CMT2 | Crimella 2012^16^ |  |
| c.710A>T | p.E237V | Motor | West syndrome and severe global developmental delay | Fukuoka 2021^29^ |  |
| c.728G>A | p.G243E | Motor | SPG10 | Méreaux 2022^7^ |  |
| c.745C>G | p.L249V | Motor | SPG10 | Lynch 2016^30^ |  |
| c.746T>C | p.L249P | Motor | SPG10 | Perić 2022^31^ |  |
| c.751G>A | p.E251K | Motor | SPG10 | Goizet 2009^2^ Neveling 2013^23^  Iqbal 2017^32^  Méreaux 2022^7^ |  |
| c.759G>T | p.K253N | Motor | SPG10 | Schüle 2008^3^ |  |
| c.763A>G | p.I255V | Motor | SPG10 | Méreaux 2022^7^ |  |
| c.765C>G | p.I255M | Motor | SPG10 | Citrigno 2018^33^ Méreaux 2022^7^ |  |
| c.767A>G | p.N256S | Motor | SPG10 | Reid 2002^34^  Andréasson 2019^35^  Méreaux 2022^7^ |  |
| c.768_770delCAA | p.N256del | Motor | SPG10 | Schüle 2008^3^ |  |
| c.771G>C | p.K257N | Motor | SPG10 | Goizet 2009^2^ |  |
| c.773C>T | p.S258L | Motor | SPG10 | López 2015^36^ |  |
| c.776T>A | p.L259Q | Motor | SPG10 + congenital deafness | Muglia 2014^37^ |  |
| c.785T>C | p.L262P | Motor | SPG10, CMT2 | Dohrn 2017^38^ |  |
| c.802G>A | p.A268T | Motor | Adult-onset distal spinal muscular atrophy | de Fuenmayor-Fernández de la Hoz 2019^39^ |  |
| c.827A>G | p.Y276C | Motor | SPG10 | Blair 2006^40^ |  |
| c.833C>T | p.P278L | Motor | SPG10 | López 2015^36^ Morais 2017^13^ Méreaux 2022^7^ |  |
| c.838C>T | p.R280C^a^ | Motor | SPG10, CMT2 | Fichera 2004^41^ Goizet 2009^2^ Liu 2014^19^ Hsu 2019^42^ Méreaux 2022^7^ |  |
| c.839G>A | p.R280H | Motor | SPG10, CMT2 | Goizet 2009^2^ Liu 2014^19^ Morais 2017^13^ Nam 2018^20^ Méreaux 2022^7^ Panwala 2022^43^ |  |
| c.839G>T | p.R280L | Motor | SPG10 | Goizet 2009^2^ |  |
| c.854C>T | p.T285I | Motor | SPG10, CMT | Bacquet 2018^5^ Méreaux 2022^7^ |  |
| c.868G>C | p.D290H | Motor | SPG10 | Morais 2017^13^ |  |
| c.889C>T | p.R297W | Motor | SPG10 | Méreaux 2022^7^ |  |
| c.967C>T | p.R323W | Motor | SPG10 | Rinaldi 2015^44^ Andréasson 2019^35^  Méreaux 2022^7^ |  |
| c.1022A>G | p.Q341R | Stalk | SPG10 | Giordani 2021^45^ |  |
| c.1082C>T | p.A361V | Stalk | SPG10, primary progressive multiple sclerosis | Lo Giudice 2006^46^ Jia 2018^47^2023-10-12 12:06:00 PM |  |
| c.1086G>C | p.K362N | Stalk | SPG10 | Guinto 2017^48^ |  |
| c.1463T>G | p.L488R | Stalk | ALS | Grassano 2022^6^ |  |
| c.1673T>C | p.L558P | Stalk | CMT | Nam 2018^20^ |  |
| c.2263G>A | p.E755K | Stalk | SPG10, ALS | Crimella 2012^16^ Grassano 2022^6^ |  |
| c.2290C>T | p.Q764* | Stalk | CMT | Hsu 2019^42^ |  |
| c.2539-605_*36 + 211del | p.L847_S1032delins33 | Stalk | CMT | Pyromali 2021^1^ |  |
| c.2590C>T | p.R864* | Stalk | SPG10 | Lynch 2016^30^ |  |
| c.2720A>T | p.K907M | Tail | Leber optic neuropathy | Pandya 2022^49^ |  |
| c.2757delC | p.K920Nfs*128 | Tail | ALS | Grassano 2022^6^ |  |
| c.2854delC | p.Q952Rfs*96 | Tail | NEIMY | Duis 2016^50^ |  |
| c.2922delC | p.C975Vfs*73 | Tail | NEIMY | Rydzanicz 2017^51^ |  |
| c.2934delG | p.S978Lfs*70 | Tail | NEIMY | DaRe 2013^52^  Duis 2016^50^ |  |
| c.2987delA | p.D996fs | Tail | ALS | Nicolas 2018^53^ |  |
| c.2990delA | p.N997fs | Tail | ALS | Nicolas 2018^53^ |  |
| c.2993-14G>T | Exon 27 skipping and frameshift (p.N999Vfs*40,^b^ ΔExon27) | Tail | ALS/FTD | Saez-Atienzar 2020^54^ |  |
| c.2993-3C>A | Exon 27 skipping and frameshift (p.N999Vfs*40,^b^ ΔExon27) | Tail | ALS | Nakamura 2021^55^ |  |
| c.2993-3C>T | Exon 27 skipping and frameshift (p.N999Vfs*40,^b^ ΔExon27) | Tail | ALS | Nicolas 2018^53^ Gu 2018^56^ |  |
| c.2993-1G>A | Exon 27 skipping and frameshift (p.N999Vfs*40,^b^ ΔExon27) | Tail | ALS | Brenner 2018^57^  Nicolas 2018^53^ Zhang 2019^58^ | predicted to create a new AS site: p.G998Efs*50 |
| c.2996delA | p.N999fs | Tail | ALS | Nicolas 2018^53^ |  |
| c.2999delC | p.T1001Qfs | Tail | ALS | He 2020^22^ |  |
| c.3005A>G | p.(D1002G) | Tail | ALS | Tunca 2020^59^ | predicted to affect splicing: p.D1002Gfs*41 |
| c.3017A>G | p.(N1006S) | Tail | ALS | This study | predicted to affect splicing (p.N999Vfs*40^b^ and/or p.N1006Rfs*41) |
| c.3019A>G | p.R1007G | Tail | ALS | Brenner 2018^57^ Nicolas 2018^53^ | predicted to affect splicing |
| c.3020G>A | p.R1007K | Tail | ALS (+ signs of SPG10 and CMT) | Nicolas 2018^53^ Dulski 2023^60^ | predicted to affect splicing |
| c.3020+1G>A | Exon 27 skipping and frameshift (p.N999Vfs*40,^b^ ΔExon27) | Tail | ALS | Brenner 2018^57^ Nicolas 2018^53^ Naruse 2021^61^ |  |
| c.3020+2T>A | Exon 27 skipping and frameshift (p.N999Vfs*40,^b^ ΔExon27) | Tail | ALS | Nicolas 2018^53^ Gu 2018^56^ |  |
| c.3020+2T>C | Exon 27 skipping and frameshift (p.N999Vfs*40,^b^ ΔExon27) | Tail | ALS | Brenner 2018^57^ |  |
| c.3020+3A>G | Exon 27 skipping and frameshift (p.N999Vfs*40,^b^ ΔExon27) | Tail | ALS (mixed with SPG10 and cerebellar ataxia) | Nicolas 2018^53^ Faruq 2019^62^ Naruse 2021^61^ |  |
| c.3020+3A>T | Exon 27 skipping and frameshift (p.N999Vfs*40,^b^ ΔExon27) | Tail | ALS (mixed with SPG10 and cerebellar ataxia) | Faruq 2019^62^ |  |

^a^Mutation characterised as p.R280S by Ebbing *et al.*^63^ and Dutta *et al.*^64^ to avoid *in vitro* oxidation.

^b^Previously referred to as p.N999Vfs*39, here described as p.N999Vfs*40 according to current HGVS recommendations for sequence variant nomenclature (https://varnomen.hgvs.org/; Mutalyzer 3: https://mutalyzer.nl).

**Supplementary Table 1 references**

1. Pyromali I, Perani A, Nizou A, et al. New structural variations responsible for Charcot-Marie-Tooth disease: The first two large KIF5A deletions detected by CovCopCan software. *Comput Struct Biotechnol J*. 2021;19:4265-4272. doi:10.1016/j.csbj.2021.07.037

2. Goizet C, Boukhris A, Mundwiller E, et al. Complicated forms of autosomal dominant hereditary spastic paraplegia are frequent in SPG10. *Hum Mutat*. 2009;30(2):E376-E385. doi:https://doi.org/10.1002/humu.20920

3. Schüle R, Kremer BPH, Kassubek J, et al. SPG10 is a rare cause of spastic paraplegia in European families. *J Neurol Neurosurg Psychiatry*. 2008;79(5):584. doi:10.1136/jnnp.2007.137596

4. Qiu Y, Zhong S, Cong L, et al. A novel KIF5A gene variant causes spastic paraplegia and cerebellar ataxia. *Ann Clin Transl Neurol*. 2018;5(11):1415-1420. doi:10.1002/acn3.650

5. Bacquet J, Stojkovic T, Boyer A, et al. Molecular diagnosis of inherited peripheral neuropathies by targeted next-generation sequencing: molecular spectrum delineation. *BMJ Open*. 2018;8(10):e021632. doi:10.1136/bmjopen-2018-021632

6. Grassano M, Calvo A, Moglia C, et al. Systematic evaluation of genetic mutations in ALS: a population-based study. *J Neurol Neurosurg Psychiatry*. 2022;93(11):1190-1193. doi:10.1136/jnnp-2022-328931

7. Méreaux JL, Banneau G, Papin M, et al. Clinical and genetic spectra of 1550 index patients with hereditary spastic paraplegia. *Brain*. 2022;145(3):1029-1037. doi:10.1093/brain/awab386

8. van de Warrenburg BP, Schouten MI, de Bot ST, et al. Clinical exome sequencing for cerebellar ataxia and spastic paraplegia uncovers novel gene-disease associations and unanticipated rare disorders. *European Journal of Human Genetics*. 2016;24(10):1460-1466. doi:10.1038/ejhg.2016.42

9. Carosi L, Lo Giudice T, Di Lullo M, et al. Hereditary spastic paraplegia: a novel mutation and expansion of the phenotype variability in SPG10. *J Neurol Neurosurg Psychiatry*. 2015;86(6):702-704. doi:10.1136/jnnp-2014-308625

10. Kaji S, Kawarai T, Miyamoto R, et al. Late-onset spastic paraplegia type 10 (SPG10) family presenting with bulbar symptoms and fasciculations mimicking amyotrophic lateral sclerosis. *J Neurol Sci*. 2016;364:45-49. doi:10.1016/j.jns.2016.03.001

11. Elert-Dobkowska E, Stepniak I, Krysa W, et al. Next-generation sequencing study reveals the broader variant spectrum of hereditary spastic paraplegia and related phenotypes. *Neurogenetics*. 2019;20(1):27-38. doi:10.1007/s10048-019-00565-6

12. Oliveira R, Maruta C, Gil-Gouveia R. A novel KIF5A mutation identified in two-family members with spastic paraplegia type 10. *Rev Neurol (Paris)*. 2021;177(1-2):152-154. doi:10.1016/j.neurol.2020.04.026

13. Morais S, Raymond L, Mairey M, et al. Massive sequencing of 70 genes reveals a myriad of missing genes or mechanisms to be uncovered in hereditary spastic paraplegias. *European Journal of Human Genetics*. 2017;25(11):1217-1228. doi:10.1038/ejhg.2017.124

14. Collongues N, Depienne C, Boehm N, et al. Novel SPG10 mutation associated with dysautonomia, spinal cord atrophy, and skin biopsy abnormality. *Eur J Neurol*. 2013;20(2):398-401. doi:10.1111/j.1468-1331.2012.03803.x

15. Cortese A, Wilcox JE, Polke JM, et al. Targeted next-generation sequencing panels in the diagnosis of Charcot-Marie-Tooth disease. *Neurology*. 2020;94(1):e51-e61. doi:10.1212/WNL.0000000000008672

16. Crimella C, Baschirotto C, Arnoldi A, et al. Mutations in the motor and stalk domains of KIF5A in spastic paraplegia type 10 and in axonal Charcot-Marie-Tooth type 2. *Clin Genet*. 2012;82(2):157-164. doi:10.1111/j.1399-0004.2011.01717.x

17. Musumeci O, Bassi MT, Mazzeo A, et al. A novel mutation in KIF5A gene causing hereditary spastic paraplegia with axonal neuropathy. *Neurological Sciences*. 2011;32(4):665-668. doi:10.1007/s10072-010-0445-8

18. Tessa A, Silvestri G, de Leva MF, et al. A novel KIF5A/SPG10 mutation in spastic paraplegia associated with axonal neuropathy. *J Neurol*. 2008;255(7):1090-1092. doi:10.1007/s00415-008-0840-8

19. Liu YT, Laura M, Hersheson J, et al. Extended phenotypic spectrum of KIF5A mutations: from spastic paraplegia to axonal neuropathy. *Neurology*. 2014;83(7):612-619. doi:10.1212/WNL.0000000000000691

20. Nam DE, Yoo DH, Choi SS, Choi BO, Chung KW. Wide phenotypic spectrum in axonal Charcot–Marie–Tooth neuropathy type 2 patients with KIF5A mutations. *Genes Genomics*. 2018;40(1):77-84. doi:10.1007/s13258-017-0612-x

21. Cuchanski M, Baldwin KJ. Mutation in KIF5A c.610C>T causing hereditary spastic paraplegia with axonal sensorimotor neuropathy. *Case Rep Neurol*. 2018;10(2):165-168. doi:10.1159/000490456

22. He J, Liu X, Tang L, Zhao C, He J, Fan D. Whole-exome sequencing identified novel KIF5A mutations in Chinese patients with amyotrophic lateral sclerosis and Charcot-Marie-Tooth type 2. *J Neurol Neurosurg Psychiatry*. 2020;91(3):326-328. doi:10.1136/jnnp-2019-320483

23. Neveling K, Feenstra I, Gilissen C, et al. A post-hoc comparison of the utility of Sanger sequencing and exome sequencing for the diagnosis of heterogeneous diseases. *Hum Mutat*. 2013;34(12):1721-1726. doi:10.1002/humu.22450

24. Orsucci D, Petrucci L, Ienco EC, et al. Hereditary spastic paraparesis in adults. A clinical and genetic perspective from Tuscany. *Clin Neurol Neurosurg*. 2014;120:14-19. doi:10.1016/j.clineuro.2014.02.002

25. Jerath NU, Grider T, Shy ME. Progressive lower extremity weakness and axonal sensorimotor polyneuropathy from a mutation in KIF5A (c.611G>A;p.Arg204Gln). *Case Rep Genet*. 2015;2015:1-5. doi:10.1155/2015/496053

26. Lee H, La Y, Na HK, Kim H, Shin S, Choi YC. Hereditary spastic paraplegia with axonal sensorimotor polyneuropathy in a Korean family caused by pathogenic variant of KIF5A (c.611G>A). *Journal of Clinical Neurology*. 2020;16(2):347. doi:10.3988/jcn.2020.16.2.347

27. 100 000 Genomes Project Pilot Investigators, Smedley D, Smith KR, et al. 100,000 Genomes pilot on rare-disease diagnosis in health care - Preliminary report. *N Engl J Med*. 2021;385(20):1868-1880. doi:10.1056/NEJMoa2035790

28. Dellatte J, Lievens I, Wang FC. Could some mutations of the KIF5A gene be responsible for a dominant CMT2 phenotype? (Case report). *Acta Neurol Belg*. Published online April 21, 2023. doi:10.1007/s13760-023-02248-4

29. Fukuoka M, Okazaki S, Kim K, et al. Preliminary report for Epilepsia Open: A case of West syndrome with severe global developmental delay and confirmed KIF5A gene variant. *Epilepsia Open*. 2021;6(1):230-234. doi:10.1002/epi4.12431

30. Lynch DS, Koutsis G, Tucci A, et al. Hereditary spastic paraplegia in Greece: characterisation of a previously unexplored population using next-generation sequencing. *European Journal of Human Genetics*. 2016;24(6):857-863. doi:10.1038/ejhg.2015.200

31. Perić S, Marković V, Candayan A, et al. Phenotypic and genetic heterogeneity of adult patients with hereditary spastic paraplegia from Serbia. *Cells*. 2022;11(18):2804. doi:10.3390/cells11182804

32. Iqbal Z, Rydning SL, Wedding IM, et al. Targeted high throughput sequencing in hereditary ataxia and spastic paraplegia. *PLoS One*. 2017;12(3):e0174667. doi:10.1371/journal.pone.0174667

33. Citrigno L, Magariello A, Pugliese P, et al. Kinesins in neurological inherited diseases: a novel motor-domain mutation in KIF5A gene in a patient from Southern Italy affected by hereditary spastic paraplegia. *Acta Neurol Belg*. 2018;118(4):643-646. doi:10.1007/s13760-018-1039-0

34. Reid E, Kloos M, Ashley-Koch A, et al. A kinesin heavy chain (KIF5A) mutation in hereditary spastic paraplegia (SPG10). *Am J Hum Genet*. 2002;71(5):1189-1194. doi:10.1086/344210

35. Andréasson M, Lagerstedt-Robinson K, Samuelsson K, et al. Altered CSF levels of monoamines in hereditary spastic paraparesis 10. *Neurol Genet*. 2019;5(4):e344. doi:10.1212/NXG.0000000000000344

36. López E, Casasnovas C, Giménez J, Santamaría R, Terrazas JM, Volpini V. Identification of two novel KIF5A mutations in hereditary spastic paraplegia associated with mild peripheral neuropathy. *J Neurol Sci*. 2015;358(1-2):422-427. doi:10.1016/j.jns.2015.08.1529

37. Muglia M, Citrigno L, D’Errico E, et al. A novel KIF5A mutation in an Italian family marked by spastic paraparesis and congenital deafness. *J Neurol Sci*. 2014;343(1-2):218-220. doi:10.1016/j.jns.2014.05.063

38. Dohrn MF, Glöckle N, Mulahasanovic L, et al. Frequent genes in rare diseases: panel-based next generation sequencing to disclose causal mutations in hereditary neuropathies. *J Neurochem*. 2017;143(5):507-522. doi:10.1111/jnc.14217

39. de Fuenmayor-Fernández de la Hoz CP, Hernández-Laín A, Olivé M, Sánchez-Calvín MT, Gonzalo-Martínez JF, Domínguez-González C. Adult-onset distal spinal muscular atrophy: a new phenotype associated with KIF5A mutations. *Brain*. 2019;142(12):e66-e66. doi:10.1093/brain/awz317

40. Blair MA, Ma S, Hedera P. Mutation in KIF5A can also cause adult-onset hereditary spastic paraplegia. *Neurogenetics*. 2006;7(1):47-50. doi:10.1007/s10048-005-0027-8

41. Fichera M, Giudice M Lo, Falco M, et al. Evidence of kinesin heavy chain (KIF5A) involvement in pure hereditary spastic paraplegia. *Neurology*. 2004;63(6):1108-1110. doi:10.1212/01.wnl.0000138731.60693.d2

42. Hsu Y, Lin K, Guo Y, Tsai Y, Liao Y, Lee Y. Mutation spectrum of Charcot‐Marie‐Tooth disease among the Han Chinese in Taiwan. *Ann Clin Transl Neurol*. 2019;6(6):1090-1101. doi:10.1002/acn3.50797

43. Panwala TF, Garcia-Santibanez R, Vizcarra JA, Garcia AG, Verma S. Childhood-onset hereditary spastic paraplegia (HSP): a case series and review of literature. *Pediatr Neurol*. 2022;130:7-13. doi:10.1016/j.pediatrneurol.2022.02.007

44. Rinaldi F, Bassi MT, Todeschini A, et al. A novel mutation in motor domain of KIF5A associated with an HSP/axonal neuropathy phenotype. *J Clin Neuromuscul Dis*. 2015;16(3):153-158. doi:10.1097/CND.0000000000000063

45. Giordani GM, Diniz F, Fussiger H, et al. Clinical and molecular characterization of a large cohort of childhood onset hereditary spastic paraplegias. *Sci Rep*. 2021;11(1):22248. doi:10.1038/s41598-021-01635-2

46. Lo Giudice M, Neri M, Falco M, et al. A missense mutation in the coiled-coil domain of the KIF5A gene and late-onset hereditary spastic paraplegia. *Arch Neurol*. 2006;63(2):284-287. doi:10.1001/archneur.63.2.284

47. Jia X, Madireddy L, Caillier S, et al. Genome sequencing uncovers phenocopies in primary progressive multiple sclerosis. *Ann Neurol*. 2018;84(1):51-63. doi:10.1002/ana.25263

48. Guinto CO, Diarra S, Diallo S, et al. A novel mutation in KIF5A in a Malian family with spastic paraplegia and sensory loss. *Ann Clin Transl Neurol*. 2017;4(4):272-275. doi:10.1002/acn3.402

49. Pandya BU, Margolin EA, Micieli JA. Nuclear DNA mutation in KIF5A causing autosomal dominant phenotypic Leber hereditary optic neuropathy. *Journal of Neuro-ophthalmology*. Published online August 2, 2022. doi:10.1097/WNO.0000000000001699

50. Duis J, Dean S, Applegate C, et al. KIF5A mutations cause an infantile onset phenotype including severe myoclonus with evidence of mitochondrial dysfunction. *Ann Neurol*. 2016;80(4):633-637. doi:https://doi.org/10.1002/ana.24744

51. Rydzanicz M, Jagła M, Kosinska J, et al. KIF5A de novo mutation associated with myoclonic seizures and neonatal onset progressive leukoencephalopathy. *Clin Genet*. 2017;91(5):769-773. doi:10.1111/cge.12831

52. DaRe JT, Vasta V, Penn J, Tran NTB, Hahn SH. Targeted exome sequencing for mitochondrial disorders reveals high genetic heterogeneity. *BMC Med Genet*. 2013;14(1):118. doi:10.1186/1471-2350-14-118

53. Nicolas A, Kenna KP, Renton AE, et al. Genome-wide analyses identify KIF5A as a novel ALS gene. *Neuron*. 2018;97(6):1268-1283.e6. doi:10.1016/j.neuron.2018.02.027

54. Saez-Atienzar S, Dalgard CL, Ding J, et al. Identification of a pathogenic intronic KIF5A mutation in an ALS-FTD kindred. *Neurology*. 2020;95(22):1015-1018. doi:10.1212/WNL.0000000000011064

55. Nakamura R, Tohnai G, Atsuta N, et al. Genetic and functional analysis of KIF5A variants in Japanese patients with sporadic amyotrophic lateral sclerosis. *Neurobiol Aging*. 2021;97:147.e11-147.e17. doi:10.1016/j.neurobiolaging.2020.07.010

56. Gu X, Li C, Chen Y, et al. Mutation screening of the KIF5A gene in Chinese patients with amyotrophic lateral sclerosis. *J Neurol Neurosurg Psychiatry*. 2019;90(2):245-246. doi:10.1136/jnnp-2018-318395

57. Brenner D, Yilmaz R, Müller K, et al. Hot-spot KIF5A mutations cause familial ALS. *Brain*. 2018;141(3):688-697. doi:10.1093/brain/awx370

58. Zhang K, Liu Q, Shen D, et al. Mutation analysis of KIF5A in Chinese amyotrophic lateral sclerosis patients. *Neurobiol Aging*. 2019;73:229.e1-229.e4. doi:10.1016/j.neurobiolaging.2018.08.006

59. Tunca C, Şeker T, Akçimen F, et al. Revisiting the complex architecture of ALS in Turkey: Expanding genotypes, shared phenotypes, molecular networks, and a public variant database. *Hum Mutat*. 2020;41(8). doi:10.1002/humu.24055

60. Dulski J, Strongosky AJ, Al-Shaikh RH, Wszolek ZK. Expanding the spectrum of KIF5A mutations — Case report of a large kindred with familial ALS and overlapping syndrome. *Amyotroph Lateral Scler Frontotemporal Degener*. Published online January 5, 2023:1-4. doi:10.1080/21678421.2022.2164204

61. Naruse H, Ishiura H, Mitsui J, et al. Splice-site mutations in KIF5A in the Japanese case series of amyotrophic lateral sclerosis. *Neurogenetics*. 2021;22(1):11-17. doi:10.1007/s10048-020-00626-1

62. Faruq M, Kumar D, Wadhwa S, et al. Intrafamilial variable spastic paraplegia/ataxia/ALS phenotype linked to a novel KIF5A mutation. *Clin Genet*. 2019;96(3):271-273. doi:10.1111/cge.13585

63. Ebbing B, Mann K, Starosta A, et al. Effect of spastic paraplegia mutations in KIF5A kinesin on transport activity. *Hum Mol Genet*. 2008;17(9):1245-1252. doi:10.1093/hmg/ddn014

64. Dutta M, Diehl MR, Onuchic JN, Jana B. Structural consequences of hereditary spastic paraplegia disease-related mutations in kinesin. *Proc Natl Acad Sci U S A*. 2018;115(46):E10822-E10829. doi:10.1073/pnas.1810622115
