# Supplementary material for: Altered molecular and cellular mechanisms in KIF5A-associated neurodegenerative or neurodevelopmental disorders
Source: Cell Death Dis. 2024 Sep 27;15(9):692. doi: 10.1038/s41419-024-07096-5 (PMC11437142; doi:10.1038/s41419-024-07096-5)
Supplement: Supplementary file 3 — Supplementary Table 2 WORD Format [file 41419_2024_7096_MOESM3_ESM.docx]

**Supplementary Table 2 Clinical data of patients carrying the *KIF5A* variants investigated in this study**

| ***KIF5A* variant** | **p.R17Q**  **c.50G>A**  **___________________________________________________** | | | **p.R280C**  **c.838C>T**  **____________________________________________________________________________________________________________________________________________** | | | | | | | | **p.R864***  **c.2590C>T** | **p.(N1006S) c.3017A>G^a^** |
| --- | --- | --- | --- | --- | --- | --- | --- | --- | --- | --- | --- | --- | --- |
| **Patient** | **Patient 1**  **(F1-I-1)** | **Patient 2**  **(F1-II-1)** | **Patient 3**  **(F1-II-2)** | **Patient 4** | **Patient 5** | **Patient 6** | **Patient 7**  **(F2-I-1)** | **Patient 8**  **(F2-II-1)** | **Patient 9**  **(F3-II-1)** | **Patient 10** | **Patient 11** | **Patient 12** | **Patient 13** |
| **Family history (no. of affected relatives)** | AD (3) | AD (3) | AD (3) | n.a. (adopted child) | S | S | AD (2) | AD (2) | AD (3) | S | S | n.a. (adopted child) | S |
| **Sex** | F | F | M | F | F | F | M | M | F | M | M | M | F |
| **Age at onset** | 51 | 35 | n.a. | 2 | 13 | 1 | 29 | 4 | 23 | childhood | 20 | 14 | 60 |
| **Age at exam** | 80 | 50 | 38 | 31 | 50 | 16 | 44 | 15 | 26 | 26 | 32 | 19 | 61 |
| **Disease duration (yrs)** | 29 | 15 | n.a. | 29 | 37 | 22 | 15 | 11 | 3 | ~20 | 12 | 5 | deceased after 20 mo from onset |
| **Predominant phenotype** | Spastic ataxia | Spastic ataxia | Spastic paraplegia | Spastic paraplegia | Spastic paraplegia | Spastic paraplegia | CMT5 | Spastic paraplegia | Spastic paraplegia | Spastic paraplegia | CMT5 | Recurrent brachial plexopathy | ALS (classic phenotype) |
| **Additional symptoms** | Cognitive impairment, polyneuropathy | Polyneuropathy | Polyneuropathy | Polyneuropathy | Polyneuropathy | None | None | None | None | Polyneuropathy, low back pain | None | None | None |
| **Severity (mild/moderate/severe)** | Severe | Mild | Mild | Mild | Severe | Severe | Moderate | Mild | Moderate | Mild | Moderate | Mild | Severe |
| **Symptom at onset** | Imbalance, LL weakness | Imbalance, muscle weakness | Mild dysarthria | Motor developmental delay | Gait difficulty | Cognitive and motor delay | Gait difficulty | Toe walking, foot intrarotation (R>L), frequent falls | Gait difficulty | Gait difficulty, mild imbalance | Foot drop | Muscle weakness and pain at right shoulder | UL proximal weakness |
| **Gait features** | Spastic-ataxic gait; needs bilateral walking aids (or walker) | Ataxic gait, broad based | Normal gait, intrarotated right foot | Imbalance, difficult tandem gait | Spastic-ataxic gait; needs bilateral walking aids (or walker) | Spastic gait, intrarotated feet | Spastic gait, with walking aids | Spastic gait | Spastic | Spastic | Ataxic, steppage | Normal | Normal |
| **LL spasticity** | No | No | No | No | Severe | Severe | ++ | + | + | ++ | No | No | No |
| **LL reflexes** | Areflexia | +++ | +++ | Knee: ++, ankle: + | +++ | +++ | ++ | ++ | ++ | +++ | Knee: ++, ankle: absent | Knee: + | ++ |
| **LL weakness** | Mild, distal | No | No | Mild | Mild, distal | No | Moderate, distal | Mild, proximal | Mild | Mild, proximal | Severe, distal | No | No |
| **LL amyotrophy** | Mild, distal | No | No | Mild, distal | n.a. | ++, distal | Moderate | No | No | Mild | Moderate, distal | No | No |
| **Babinski sign** | Yes | Yes | Yes | Yes | Yes | Yes | Yes | Yes | Yes | Yes | Yes | No | No |
| **UL spasticity** | No | No | No | No | No | No | No | No | Mild | No | No | No | No |
| **UL reflexes** | Areflexia | Areflexia | Hyporeflexia | Areflexia | +++ | ++ | Normal | Normal | ++ | Hyporeflexia | Areflexia | No | +++ |
| **UL weakness** | Mild, distal | No | No | Mild, distal | Mild, distal | No | No | No | Mild | No | Mild | Mild, right UL | ++ L, + R |
| **UL amyotrophy** | Mild, distal | No | No | Mild, distal | No | no, distonic posture | No | No | No | No | Moderate, distal | Atrophy of right shoulder girdle | ++ L, + R |
| **Sensory disturbances** | Reduced deep sensation, LL, distal | No | No | Reduced deep sensation, LL, distal, mild | Reduced deep sensation, LL, distal, mild | n.a. | No | No | No | Reduced pinprick sensation at ankle | Distal sensory loss for pinprick (up to the ankle) and vibration (at toes) | Hypoesthesia right UL, proximal | No |
| **Pes cavus/scoliosis** | Pes cavus | No | Scoliosis | Pes cavus | Pes cavus | Distal tendon retraction | Pes cavus, intrarotated | No | Pes planus | Pes cavus, hammer toes | Pes cavus, left claw hand | No | No |
| **Bladder dysfunction** | Incontinence | No | No | n.a. | Episodic incontinence | No | No | Urinary retention | No | Urgency | No | No | No |
| **Neurophysiology** | Axonal sensory- motor polyneuropathy | Mild LL polyneuropathy, abnormal MEPs | NA | Mixed sensory- motor polyneuropathy, abnormal MEPs LL | Mixed sensory- motor polyneuropathy, severe, abnormal LL SEPs | n.a. | Mixed LL sensory- motor polyneuropathy | n.a. | n.a. | Axonal sensory- motor polyneuropathy | Asymmetrical axonal sensory- motor polyneuropathy; abnormal VEPs, MEPs, and BAEPs | EMG: clear neurogenic pattern in C5 muscles; very mild neurogenic changes in LL distal muscles | Symmetrical acute and chronic denervation at four limbs, bilateral carpal tunnel  syndrome, |
| **Cerebral MRI** | Normal | Normal | NA | Slight thinning of corpus callosum, slight vermian hypotrophy | Periventricular white matter lesions | Normal | Normal | Periventricular white matter hyperintensities | Normal | Normal | Mild ventricular enlargement | n.a. | Normal (microbleeding in right temporo- parietal region) |
| **Spinal cord MRI** | n.a. | Normal | n.a. | n.a. | Mild thinning of cervical and thoracic spinal cord | n.a. | Normal | n.a. | Normal | n.a. | n.a. | Normal brachial plexus and cervical spine | Normal |
| **Additional features** | Dysarthria, dysphagia, vertical gaze ophthalmoplegia, diabetes | Saccadic dysmetria, dysphagia | None | Saccadic pursuit, Romberg | None | Cognitive deficits, language defect, increased lactic acid levels | Relative-reported outcomes: wheelchair bound since age 45 yrs, mild cognitive impairment,  sphincter disturbances | Mild speech developmental delay, mild cognitive impairment | Affected relatives (mother, 1 maternal uncle, 1 maternal aunt) wheelchair bound since 5th decade | Episodic fecal incontinence | Mild intellectual disability | Bipolar disorder; borderline intellectual ability (IQ=78) | Fasciculations UL and LL |

^a^ Variant with predicted effect on splicing, not experimentally proven. Predicted both to attenuate/abolish the constitutive donor site of intron 27 and to create a new GT donor site within exon 27 at position c.3017_3018, which would generate two possible KIF5A forms: p.N999Vfs*40 and/or p.N1006Rfs*41 (Figure 2B).

AD = autosomal dominant; BAEPs = brainstem auditory evoked potentials; CMT5 = Charcot-Marie-Tooth disease type 5 (polyneuropathy with pyramidal signs); EMG = electromyography; IQ = intelligence quotient; LL = lower limb; MEPs = motor evoked potentials; n.a. = not available; S = sporadic; SEPs = somatosensory evoked potentials; UL = upper limb; VEPs = visual evoked potentials
