# Supplementary material for: Altered molecular and cellular mechanisms in KIF5A-associated neurodegenerative or neurodevelopmental disorders
Source: Cell Death Dis. 2024 Sep 27;15(9):692. doi: 10.1038/s41419-024-07096-5 (PMC11437142; doi:10.1038/s41419-024-07096-5)
Supplement: Supplementary file 4 — Supplementary Table 3 WORD Format [file 41419_2024_7096_MOESM4_ESM.docx]

**Supplementary Table 3 Quantification of the percentage of HDAC6- or GFP-G3BP1-positive KIF5A inclusions**

| **% HDAC6-positive inclusions** | | | |
| --- | --- | --- | --- |
| KIF5A: | WT | N999Vfs*40 | C975Vfs*73 |
| Field 1 | 0 | 0 | 78.57 |
| Field 2 | 0 | 0 | 90.91 |
| Field 3 | 0 | 0 | 69.23 |
| Field 4 | 0 | 0.04 | 58.82 |
| Field 5 | 0 | 0 | 58.82 |
| **Mean** | **0** | **0.01** | **71.27** |
| Tot. inclusions | 0 | 88 | 97 |
| **% GFP-G3BP1-positive inclusions** | | | |
| KIF5A: | WT | N999Vfs*40 | C975Vfs*73 |
| Field 1 | 0 | 0 | 30.00 |
| Field 2 | 0 | 0 | 27.27 |
| Field 3 | 0 | 0 | 28.00 |
| Field 4 | 0 | 0 | 17.39 |
| Field 5 | 0 | 0 | 20.00 |
| Field 6 | 0 | 0 | 12.50 |
| **Mean** | **0** | **0** | **22.53** |
| Tot. inclusions | 0 | 94 | 100 |
