# Supplementary material for: Altered molecular and cellular mechanisms in KIF5A-associated neurodegenerative or neurodevelopmental disorders
Source: Cell Death Dis. 2024 Sep 27;15(9):692. doi: 10.1038/s41419-024-07096-5 (PMC11437142; doi:10.1038/s41419-024-07096-5)
Supplement: Supplementary file 5 — Supplementary Table 4 WORD Format [file 41419_2024_7096_MOESM5_ESM.docx]

**Supplementary Table 4 List of antibodies used in this work**

| **Antibody** | **Application and dilution** | **Source** |
| --- | --- | --- |
| Mouse monoclonal anti-NKHC1 (KIF5A) | WB 1:1,000 | Santa Cruz Biotechnology, sc-374666 |
| Mouse monoclonal anti-FLAG M2 | WB 1:1,000  IF 1:500 | Sigma-Aldrich, F1804 |
| Mouse monoclonal anti-GFP | WB 1:1,000 | Immunological Sciences, MAB-94345 |
| Rabbit polyclonal anti-p62/SQSTM1 | WB 1:2,000 | Sigma-Aldrich, P0067 |
| Rabbit polyclonal anti-LC3 | WB 1:2,000 | Sigma-Aldrich, L8918 |
| Mouse monoclonal anti-ubiquitinated proteins (FK2) | IF 1:500 | Merck, 04-263 |
| Rabbit monoclonal anti-HDAC6 | IF 1:500 | Abcam, ab117516 |
| Mouse monoclonal anti-BAG1 | IF 1:50 | Santa Cruz Biotechnology, sc-376848 |
| Mouse monoclonal anti-α-tubulin | WB 1:2,000 | Sigma-Aldrich, T6199 |
| Mouse monoclonal anti-GAPDH | WB 1:3,000 | Immunological Sciences, MAB-10578 |
| Rabbit monoclonal anti-β3-tubulin | IF 1:400 | Cell Signaling Technologies, 5568 |
| Alexa Fluor® 594 goat anti-mouse IgG | IF 1:1,000 | Thermo Fisher Scientific, A11020 |
| Alexa Fluor® 594 goat anti-rabbit IgG | IF 1:1,000 | Thermo Fisher Scientific, A11072 |
| Goat polyclonal anti-mouse IgG | WB 1:10,000 | Jackson ImmunoResearch, 115-035-003 |
| Goat polyclonal anti-rabbit IgG | WB 1:10,000 | Jackson ImmunoResearch, 115-035-003 |
