# Supplementary material for: Altered molecular and cellular mechanisms in KIF5A-associated neurodegenerative or neurodevelopmental disorders
Source: Cell Death Dis. 2024 Sep 27;15(9):692. doi: 10.1038/s41419-024-07096-5 (PMC11437142; doi:10.1038/s41419-024-07096-5)
Supplement: Supplementary file 6 — Supplementary Table 5 WORD Format [file 41419_2024_7096_MOESM6_ESM.docx]

**Supplementary Table 5 List of plasmids used in this work**

| **Plasmid** | **Applications** | **Source** |
| --- | --- | --- |
| pKIF5A | WT or mutant KIF5A overexpression | Eurofins Genomics |
| pGFP-KIF5A | WT or mutant GFP-KIF5A overexpression | Insertion cloning |
| pFLAG-KIF5A | WT or mutant FLAG-KIF5A overexpression | Insertion cloning |
| pCDNA3.1 | Transfection mock | Invitrogen, V790-20 |
| pEGFP-N1 | Transfection efficiency evaluation  Transfection control for IF | Clontech, U55762 |
| pDsRed2-Mito | Mitochondrial reporter | Takara, 632421 |
| pDEST-mCherry-p62 | SQSTM1/p62 labelling | Prof. Terje Johansen (The Artic University of Norway, Norway) |
| Ub-R-YFP | 26S proteasome activity reporter | Prof. Nico Dantuma (Karolinska Institute, Sweden) |
| pFLAG-VCP | Co-immunoprecipitation contol | Prof. J.P. Taylor (St. Jude Children's Research Hospital, Memphis, TN, USA) |
| pEGFP-G3BP1 | G3BP1 labelling | Dr. Anthony K. L. Leung (Bloomberg School of Public Health, Johns Hopkins University, Baltimore, MD, USA) |

WB: western blot; IF: immunofluorescence
