# Supplementary material for: Altered molecular and cellular mechanisms in KIF5A-associated neurodegenerative or neurodevelopmental disorders
Source: Cell Death Dis. 2024 Sep 27;15(9):692. doi: 10.1038/s41419-024-07096-5 (PMC11437142; doi:10.1038/s41419-024-07096-5)

Figure 5B

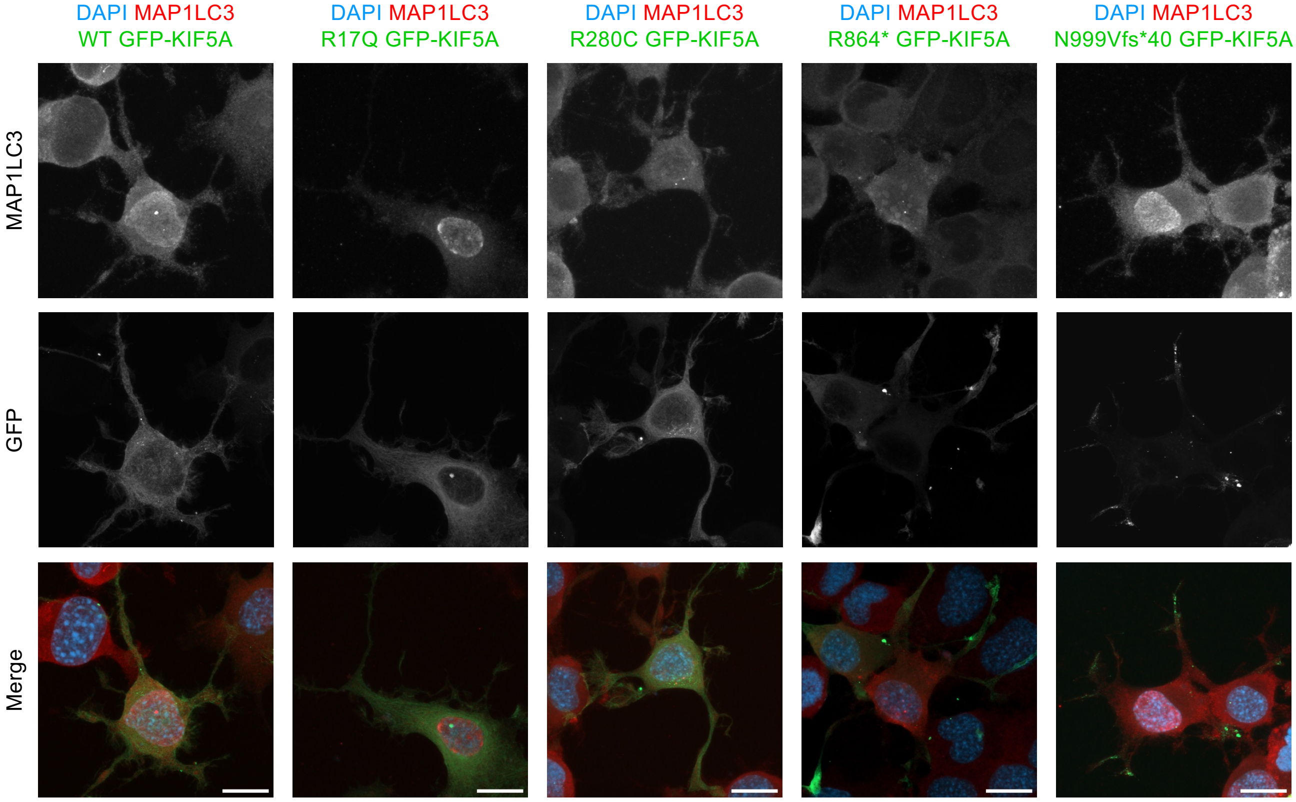

Figure 5C

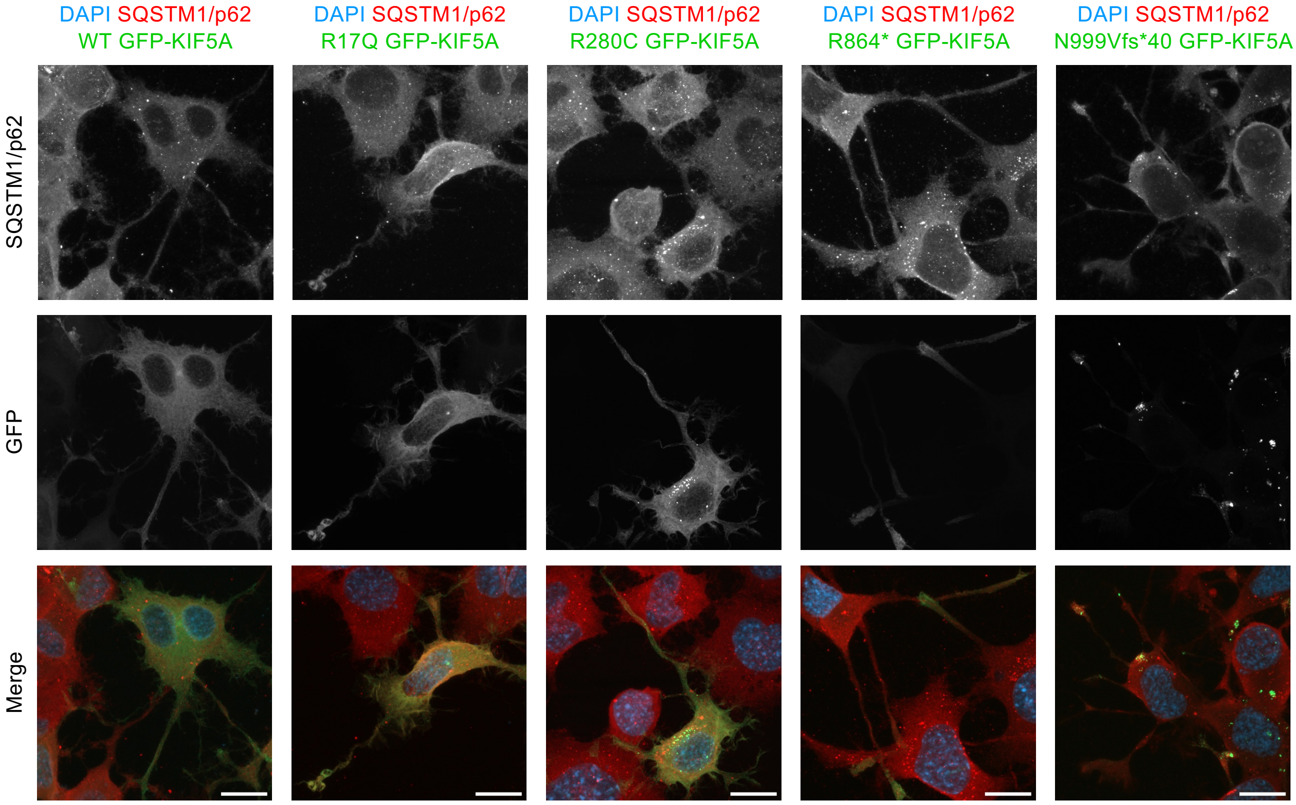

Figure 7A

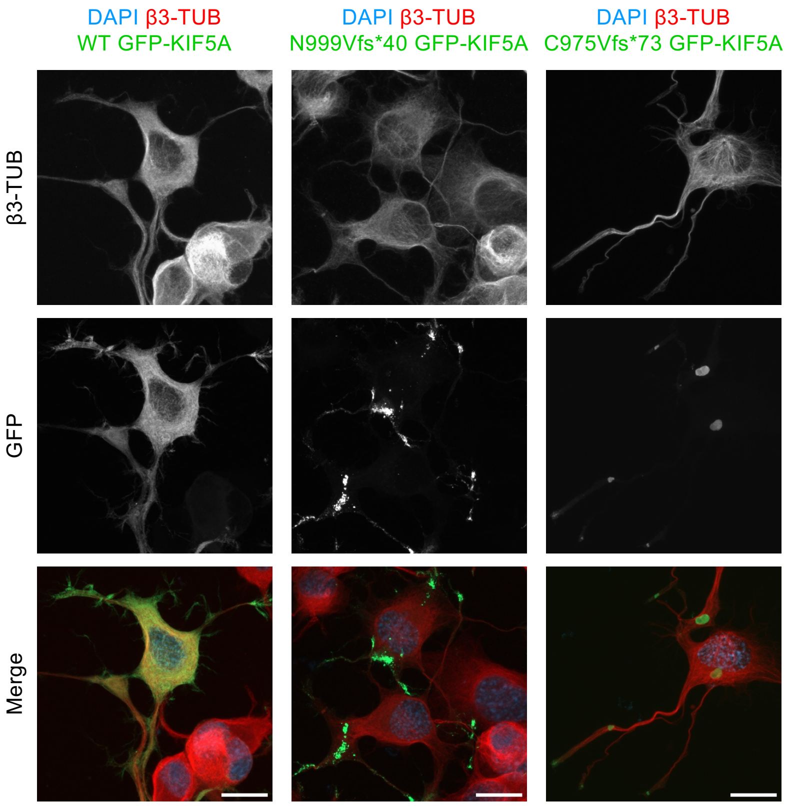

Figure 7B

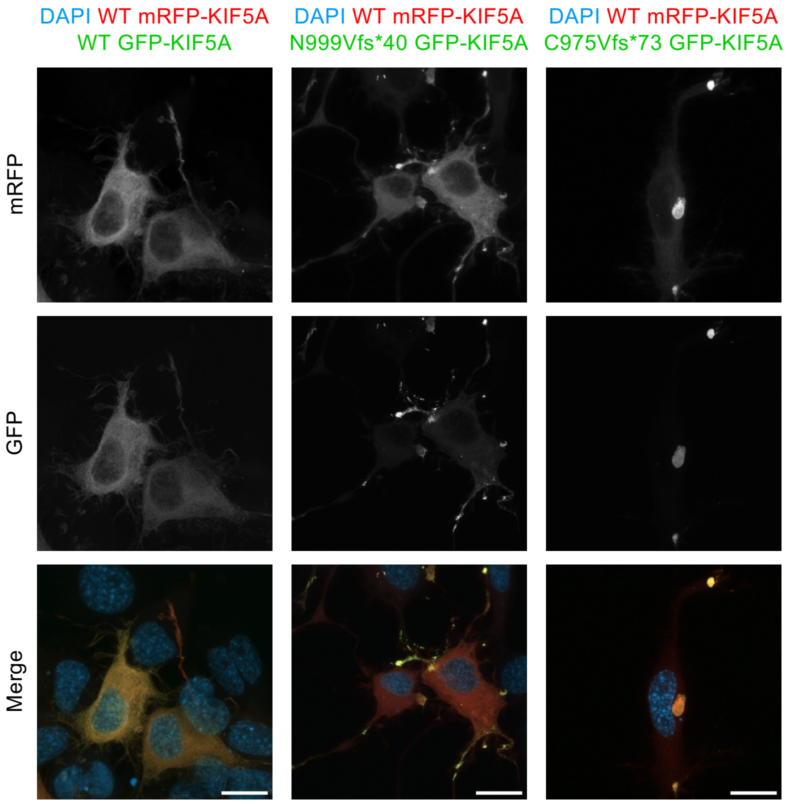

Figure 7C

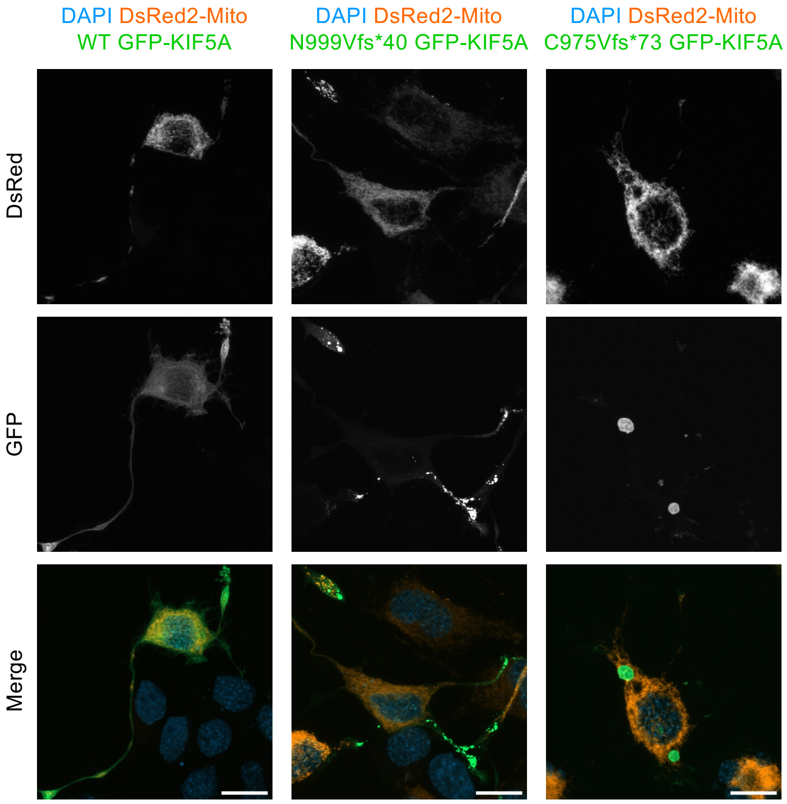

Figure 7E

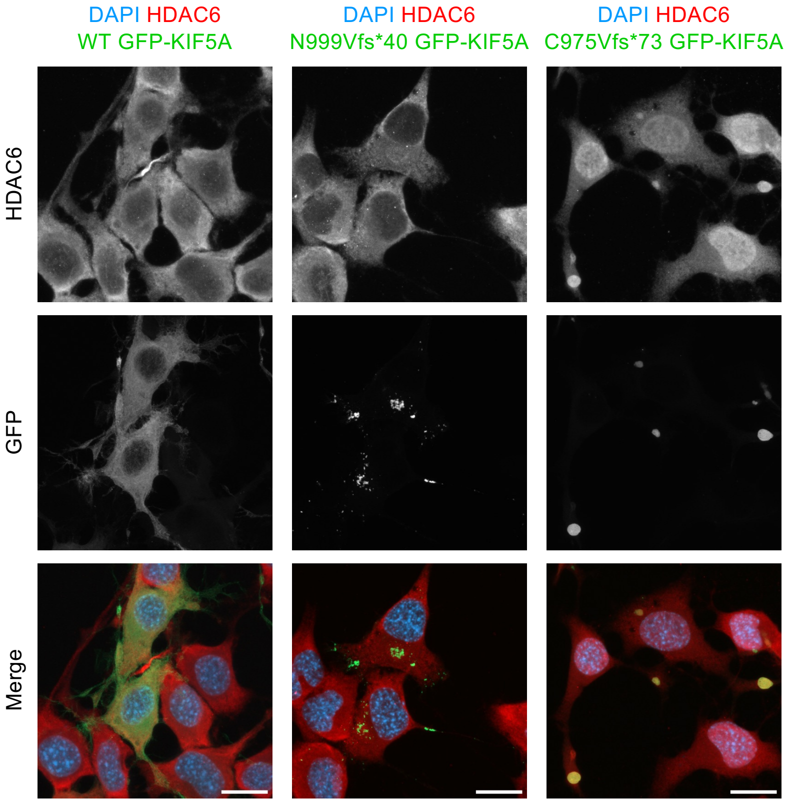

Representative images of the quantification in Supplementary Figure 3B

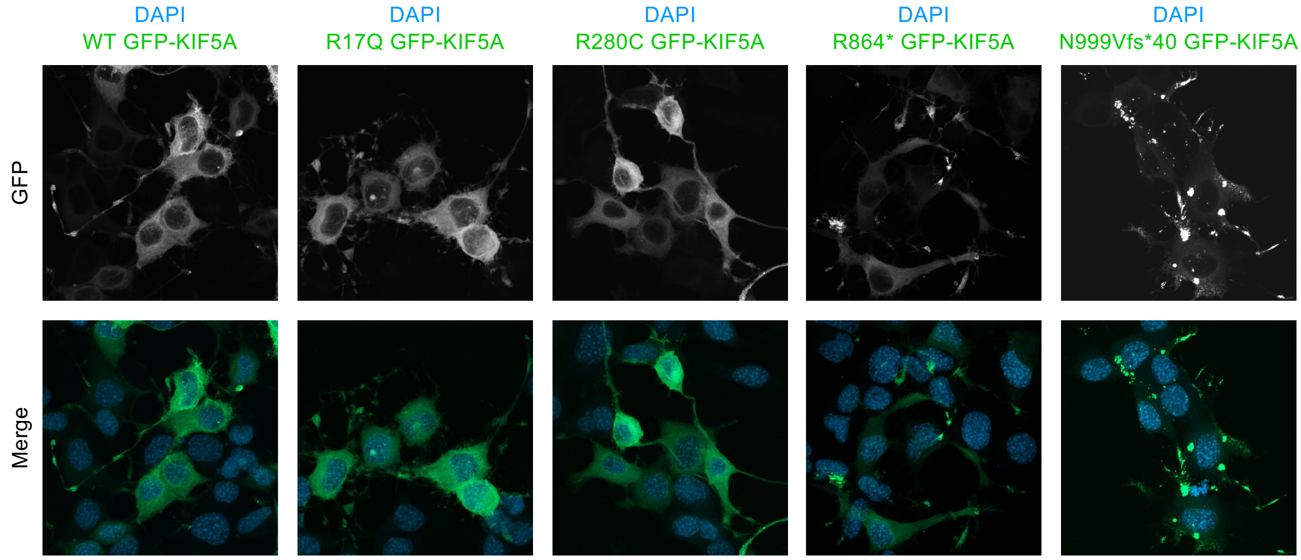

Representative images of the quantification in Supplementary Figure 4A

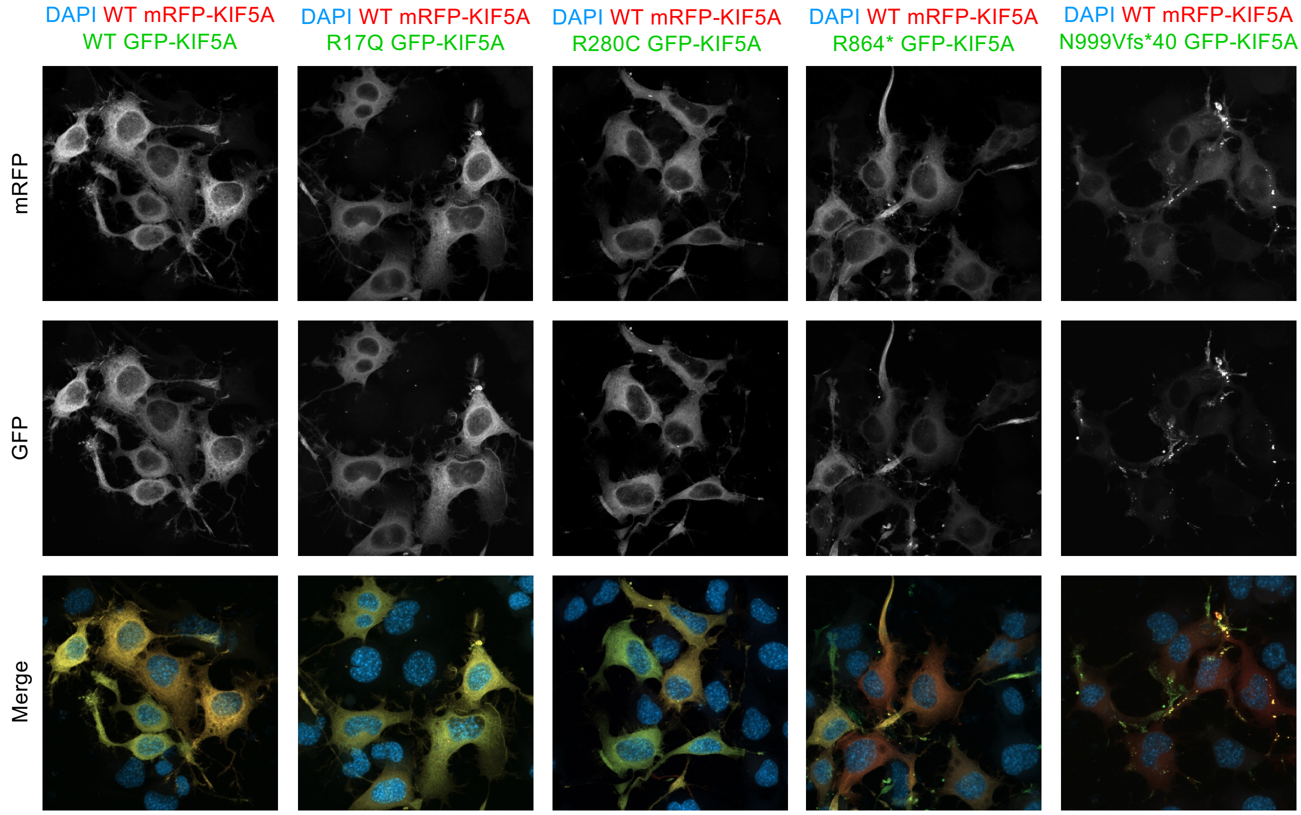

Supplementary Figure 8B

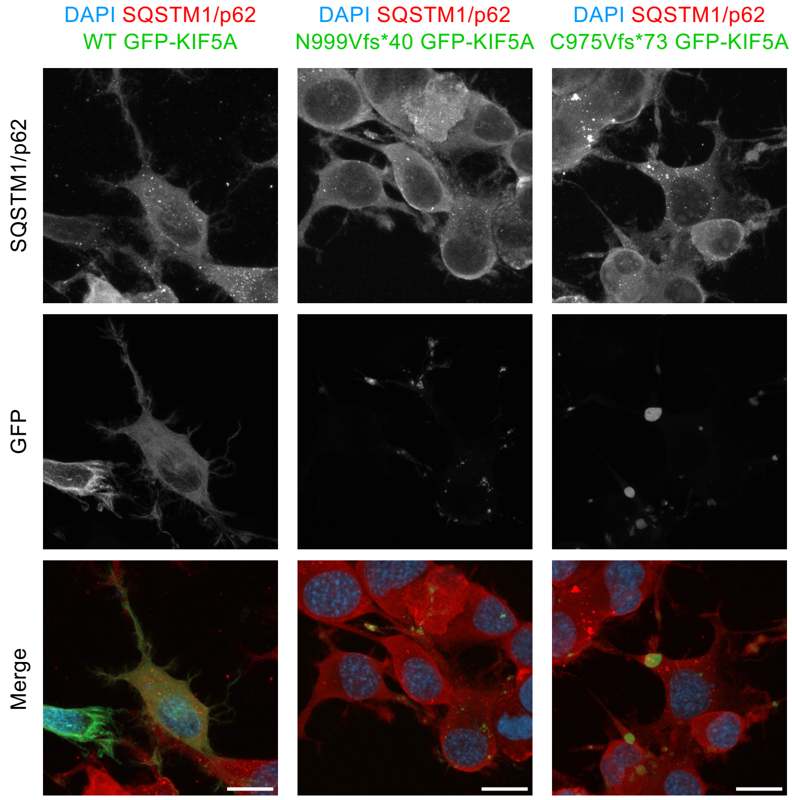

Supplementary Figure 8D

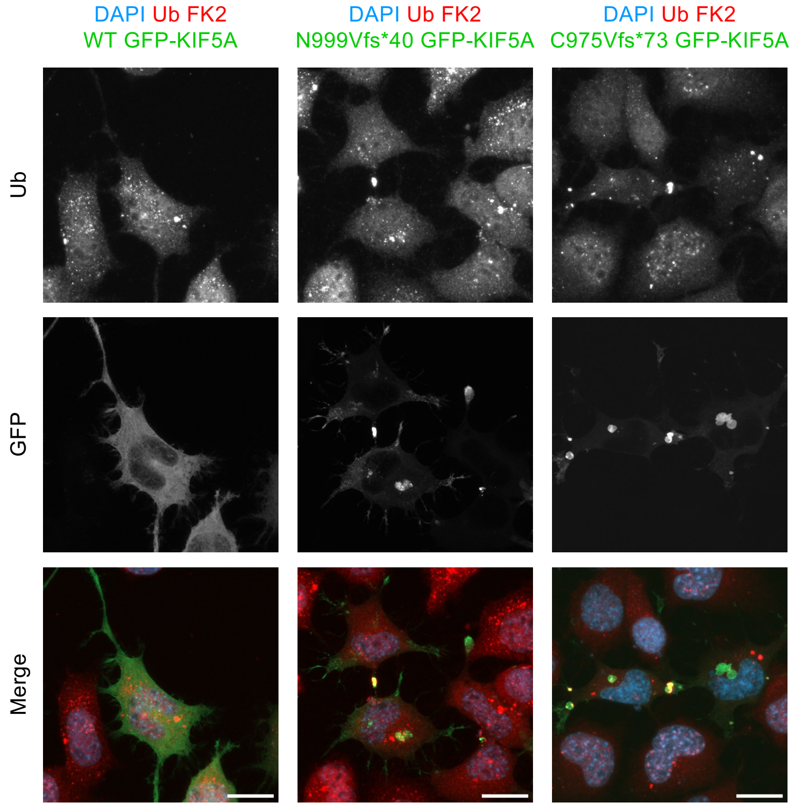

Supplementary Figure 8D

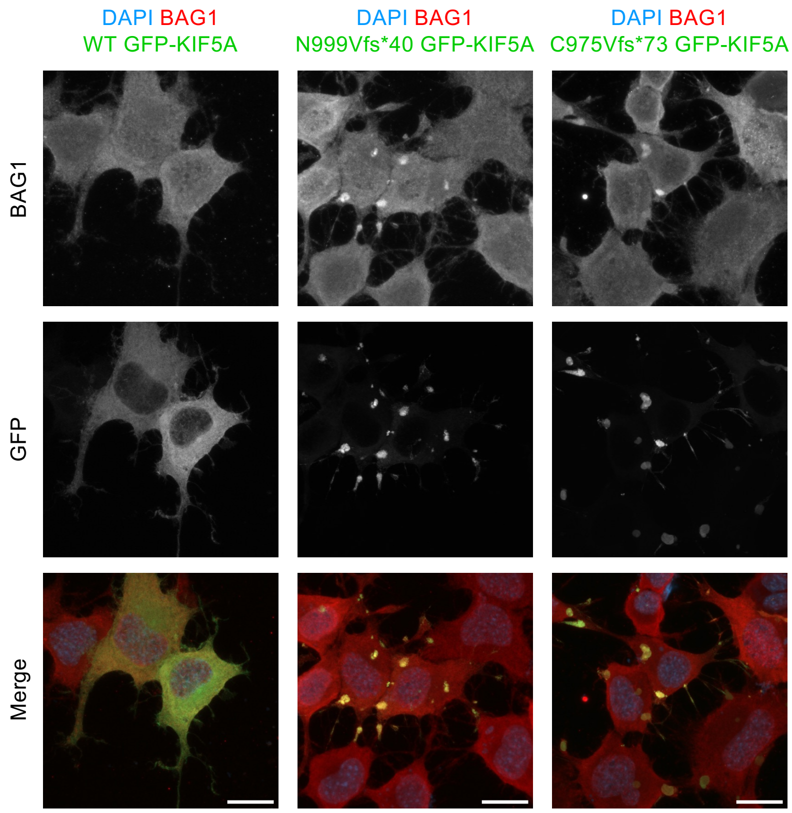

Supplementary Figure 9A

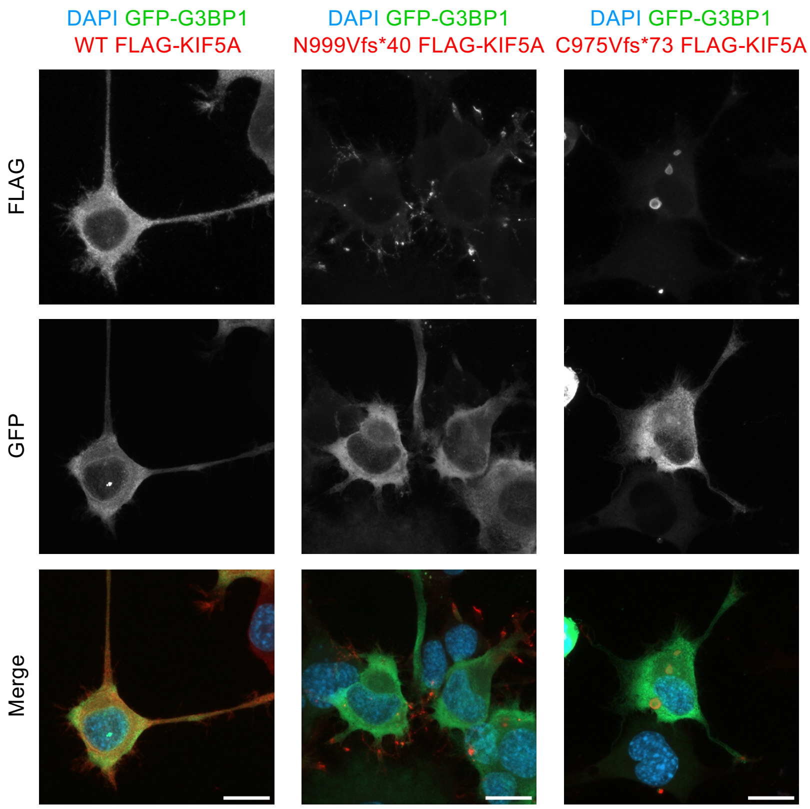

Supplement: Supplementary file 7 — Supplementary immunofluorescence images [file 41419_2024_7096_MOESM7_ESM.pdf]
